# *Adamtsl2* deletion results in bronchial fibrillin microfibril accumulation and bronchial epithelial dysplasia – a novel mouse model providing insights into geleophysic dysplasia

**DOI:** 10.1242/dmm.017046

**Published:** 2015-05-01

**Authors:** Dirk Hubmacher, Lauren W. Wang, Robert P. Mecham, Dieter P. Reinhardt, Suneel S. Apte

**Affiliations:** ^1^ Department of Biomedical Engineering, Lerner Research Institute, Cleveland Clinic, Cleveland, OH 44195, USA; ^2^ Department of Cell Biology and Physiology, Washington University School of Medicine, Saint Louis, MO 63110, USA; ^3^ Department of Anatomy and Cell Biology and Faculty of Dentistry, McGill University, 3640 University Street, Montreal, Quebec, CanadaH3A 0C7

**Keywords:** ADAMTS-like protein, Connective tissue disorder, Extracellular matrix, Fibrillin microfibril, Lung development

## Abstract

Mutations in the secreted glycoprotein ADAMTSL2 cause recessive geleophysic dysplasia (GD) in humans and Musladin–Lueke syndrome (MLS) in dogs. GD is a severe, often lethal, condition presenting with short stature, brachydactyly, stiff skin, joint contractures, tracheal-bronchial stenosis and cardiac valve anomalies, whereas MLS is non-lethal and characterized by short stature and severe skin fibrosis. Although most mutations in fibrillin-1 (FBN1) cause Marfan syndrome (MFS), a microfibril disorder leading to transforming growth factor-β (TGFβ) dysregulation, domain-specific FBN1 mutations result in dominant GD. ADAMTSL2 has been previously shown to bind FBN1 and latent TGFβ-binding protein-1 (LTBP1). Here, we investigated mice with targeted *Adamtsl2* inactivation as a new model for GD (*Adamtsl2*^−/−^ mice). An intragenic *lacZ* reporter in these mice showed that ADAMTSL2 was produced exclusively by bronchial smooth muscle cells during embryonic lung development. *Adamtsl2*^−/−^ mice, which died at birth, had severe bronchial epithelial dysplasia with abnormal glycogen-rich inclusions in bronchial epithelium resembling the cellular anomalies described previously in GD. An increase in microfibrils in the bronchial wall was associated with increased FBN2 and microfibril-associated glycoprotein-1 (MAGP1) staining, whereas LTBP1 staining was increased in bronchial epithelium. ADAMTSL2 was shown to bind directly to FBN2 with an affinity comparable to FBN1. The observed extracellular matrix (ECM) alterations were associated with increased bronchial epithelial TGFβ signaling at 17.5 days of gestation; however, treatment with TGFβ-neutralizing antibody did not correct the epithelial dysplasia. These investigations reveal a new function of ADAMTSL2 in modulating microfibril formation, and a previously unsuspected association with FBN2. Our studies suggest that the bronchial epithelial dysplasia accompanying microfibril dysregulation in *Adamtsl2*^−/−^ mice cannot be reversed by TGFβ neutralization, and thus might be mediated by other mechanisms.

## Abstract (precis)

**Summary:** The extracellular protein ADAMTSL2 is a crucial regulator of microfibril composition in the extracellular matrix of bronchial smooth muscle cells and influences bronchial epithelial function.

## INTRODUCTION

Fibrillin (FBN) microfibrils are extracellular matrix (ECM) structures recognizable in electron microscopy as 10- to 12-nm fibrils, which typically form bundles. They are widely distributed and frequently found in association with elastic fibers (Keene et al., 1991). In the ocular zonule and dermis, microfibrils perform a mechanical role (Pessier and Potter, 1996; Shi et al., 2013). In several other contexts, they are recognized as playing a crucial role in regulating the spatial distribution and activation of members of the transforming growth factor-β (TGFβ) family by directly binding to the large latent complexes of transforming growth factor-β (TGFβ) or to the prodomains of bone morphogenetic proteins (BMPs) (Sengle et al., 2008; Massam-Wu et al., 2010; Zilberberg et al., 2012). In mice with mutant *Fbn1*, TGFβ dysregulation has been identified as being a major mechanism of the observed heart, skeletal muscle, ascending aorta and lung anomalies (Neptune et al., 2003; Habashi et al., 2006, 2011; Cohn et al., 2007; Holm et al., 2011). Analysis of mice with targeted inactivation of *Fbn1* or *Fbn2* has demonstrated that they have a crucial role in regulating both TGFβ and BMPs (Arteaga-Solis et al., 2001; Nistala et al., 2010a,b). Whereas humans have three fibrillins, mice have only two, with expression of *Fbn2* dominating the embryonic period. A switch in expression from *Fbn1* to *Fbn2* occurs perinatally, and, subsequently, *Fbn1* expression dominates adulthood (Zhang et al., 1994; Mariencheck et al., 1995; Corson et al., 2004).

*FBN1* mutations typically cause Marfan syndrome (MFS, incidence 2-3 in 10,000), a serious connective tissue disorder affecting the cardiovascular and musculoskeletal systems, the eyes and the lungs (Robinson et al., 2006). Molecular mechanisms in MFS involve aberrant TGFβ signaling and structural anomalies, for example defective ciliary zonules in the eye, defects of the elastic fiber system in the aorta and altered mechanotransduction in cardiomyocytes (Wheatley et al., 1995; Traboulsi et al., 2000; Brooke et al., 2008; Doyle et al., 2012; Cook et al., 2014). Some *FBN1* mutations cause dominant geleophysic dysplasia (GD), a rare condition with musculoskeletal features (short stature, short digits, and stiff joints) that are broadly the opposite of MFS (Le Goff et al., 2011). The GD-causing mutations all localize to the TGFβ-binding protein-like domain 5 (TB5) of FBN1 (Le Goff et al., 2011). GD is also characterized by progressive tracheal-bronchial and cardiac valve fibrosis, which are implicated in early lethality and whose pathobiology is poorly understood (Le Goff and Cormier-Daire, 2009). Recessive GD is caused by mutations in *ADAMTSL2* (Le Goff et al., 2008). *ADAMTSL2* encodes a secreted glycoprotein previously shown to bind FBN1 and latent TGFβ-binding protein-1 (LTBP1) (Koo et al., 2007; Le Goff et al., 2008, 2011), suggesting that there is a role for ADAMTSL2 in the formation and function of microfibrils. Indeed, increased TGFβ signaling has been observed in dermal fibroblasts derived from GD patients (Le Goff et al., 2008, 2011). Using RNA *in situ* hybridization, it has been shown that murine *Adamtsl2* is expressed in the bronchial tree during embryogenesis, during skeletal myogenesis and at other sites (Koo et al., 2007).

**TRANSLATIONAL IMPACT**

**Clinical issue**

Geleophysic dysplasia (GD) is an inherited, rare and frequently lethal condition that affects the musculoskeletal, cardiac and pulmonary systems and the skin. Significant morbidity and mortality in GD result from poorly understood tracheal-bronchial occlusion, which leads to recurrent pulmonary infections and poor post-surgical recovery. Recessive and dominant GD are caused by mutations in the secreted extracellular matrix (ECM) proteins, ADAMTSL2 (a disintegrin and metalloproteinase with thrombospondin motifs-like 2) and fibrillin-1 (FBN1), respectively, implying a functional link between ADAMTSL2 and fibrillin microfibrils that is not fully understood, but is thought to involve transforming growth factor-β (TGFβ) dysregulation. ADAMTSL2 mutations in dogs cause Musladin-Lueke syndrome, whose hallmarks are skin fibrosis and joint contractures, but these dogs do not develop pulmonary abnormalities. In search of an alternative animal model to analyze disease mechanisms in the lung, we investigated lung development in ADAMTSL2-deficient (*Adamtsl2^−/−^*) mice.

**Results**

*Adamtsl2* was expressed specifically and dynamically by embryonic bronchial smooth muscle cells, but not by the bronchial epithelium. *Adamtsl2^−/−^* mice died at birth due to severe bronchial epithelial dysplasia, which occluded the bronchial lumen. This bronchial epithelium contained glycogen-rich inclusions that were essentially similar to cellular anomalies found in biopsies from GD-affected individuals. Bronchial epithelial dysplasia was accompanied by a profound increase in FBN2 of microfibrils and the amount of microfibril-associated glycoprotein-1 (MAGP1) associated with bronchial smooth muscle ECM. Although elevated TGFβ signaling was seen in bronchial epithelium in late gestation, TGFβ-neutralizing antibody treatment did not revert the epithelial dysplasia to normal. In addition, ADAMTSL2 was shown to bind directly to FBN2 with an affinity comparable to FBN1.

**Implications and future directions**

This study suggests that ADAMTSL2 has a major role in regulating FBN2 assembly in microfibrils. Thus, it could represent a crucial regulator of microfibril composition in the bronchial smooth muscle cell layer, and influence bronchial epithelial function through TGFβ-independent mechanisms. The *Adamtsl2^−/−^* mouse provides a new model system for the bronchial and cellular pathology of GD and identifies a previously unsuspected effect of ADAMTSL2 deficiency on FBN2 microfibrils in the embryonic period. Future studies will require the cellular mechanisms of microfibril increase and epithelial glycogen accumulation in mutant bronchi to be addressed. Because the global *Adamtsl2* inactivation leads to neonatal death, tissue specific conditional deletion will be required to determine postnatal effects of ADAMTSL2 deficiency on limbs, heart valves and skin.

Musladin-Lueke syndrome (MLS), a canine non-progressive, non-lethal condition caused by a founder Arg221Cys *ADAMTSL2* mutation in beagles (which is identical to a mutation identified in GD), also causes short stature, skin and joint fibrosis with GD, but does not give rise to cardiopulmonary problems (Bader et al., 2010; Allali et al., 2011). Thus, musculoskeletal features and fibrosis are common to both GD and MLS, but the severity and associated mortality of GD appear to directly result from the lung and/or cardiac anomalies, which are absent in MLS. MLS provides a potential model for the pathogenesis of skeletal and skin abnormalities in GD. However, owing to the availability of genetic testing, the MLS mutation will likely disappear in beagle colonies (Mellersh, 2012). In addition, logistical and economic considerations render MLS an impractical model system for GD. Given that MLS dogs do not develop the lung or cardiac anomalies observed in GD, an alternative model system is required for investigation of the pathology in these organs.

Here, we used a new mouse model of ADAMTSL2 deficiency to understand the impact of ADAMTSL2 deletion on lung development. This analysis also investigated the relationship between ADAMTSL2 and microfibrils, revealing a hitherto unsuspected impact on the FBN2 content of microfibrils. Similarities between the *Adamtsl2*^−/−^ lungs and previously published histological findings on GD suggest that this mouse model could be useful for further studies of GD pathogenesis and to evaluate potential therapeutical interventions.

## RESULTS

### Targeted deletion of *Adamtsl2* in mice leads to neonatal lethality, profound bronchial epithelial dysplasia and cellular glycogen accumulation

**Fig. 1.**
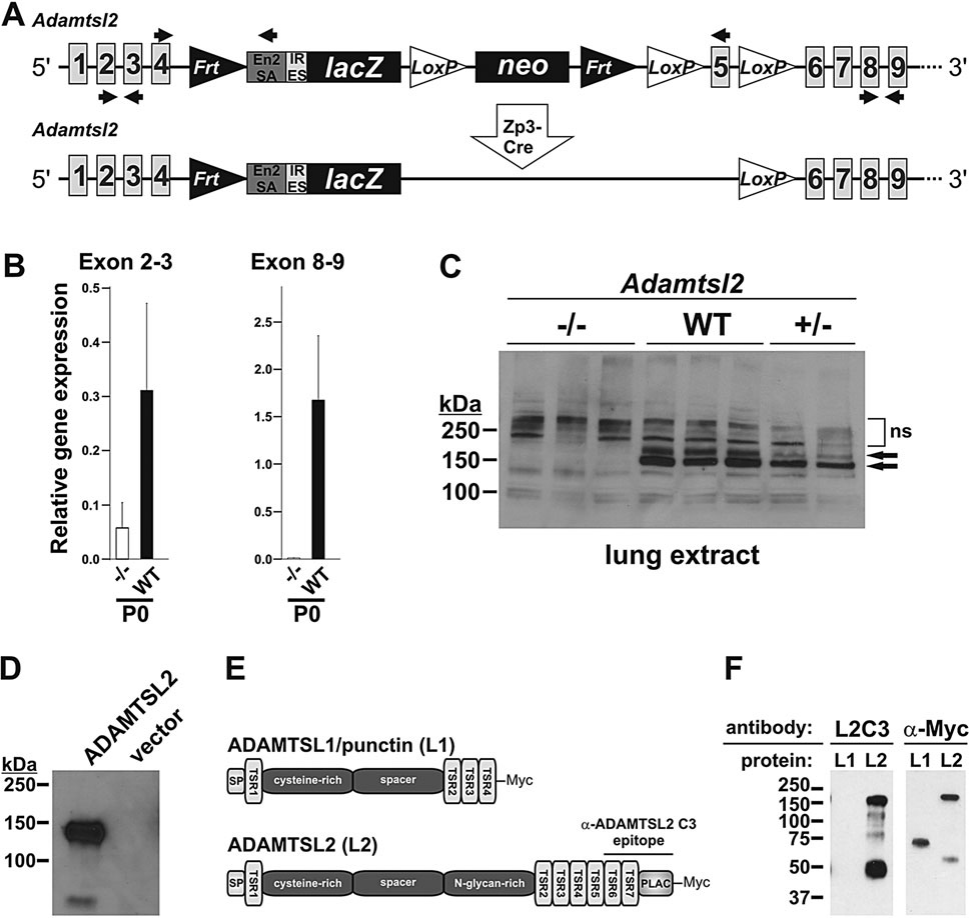
***Adamtsl2* targeting strategy and validation of inactivation.** (A) Cartoon of the gene-targeting construct (top) and following *Zp3*-Cre excision of the neomycin (*neo*) cassette (bottom), which was performed to improve β-galactosidase staining. Primers used for genotyping (top) and for qRT-PCR (bottom) are indicated as arrows. (B) *Adamtsl2* qRT-PCR was performed to verify *Adamtsl2* deletion (means±s.d., *n*=3 per genotype) using two primer pairs with RNA extracted from lung tissue prior to *Zp3*-Cre mediated *neo* excision. Note that the expression of the RNA upstream of the targeting site (exon 2-3) is reduced, presumably due to the presence of the *neo* cassette, whereas downstream primers (exon 8-9) produce no product in *Adamtsl2*^−/−^ lung. (C) Western blot of total lung protein from newborn mice probed with polyclonal antibody against the C-terminus of ADAMTSL2 (C3). 200 µg of total protein was loaded per lane. Two ADAMTSL2 bands (attributed to N-glycosylated and unmodified forms) at ∼150 kDa (arrows) are absent in protein extracts from *Adamtsl2*^−/−^ lungs and are present at reduced levels in *Adamtsl2*^+/−^ lungs (*n*=3 for WT and *Adamtsl2*^−/−^; *n*=2 for *Adamtsl2*^+/−^). (D) Conditioned medium from HEK293 cells secreting recombinant human ADAMTSL2 was used to confirm the reactivity and specificity of the anti-ADAMTSL2-C3 antibody. No reactivity was seen in the medium of empty-vector-transfected cells. (E) Cartoon of the domain organization of ADAMTSL1 (punctin-1) and ADAMTSL2. The localization of the anti-ADAMTSL2 C3 antibody epitope is indicated. (F) Western blot analysis of equal amounts of ADAMTSL1 (L1) and ADAMTSL2 (L2) detected with the anti-ADAMTSL2 antibody (L2C3, left-hand panel) or the anti-Myc (α-Myc) antibody (right-hand panel). Note that the anti-ADAMTSL2 antibody specifically recognized ADAMTSL2, whereas the α-Myc antibody recognized both proteins. The band at ∼50 kDa in the ADAMTSL2 lanes represents an ADAMTSL2 degradation product. En2SA, En2 splice acceptor; IRES, internal ribosome entry site; ns, non-specific bands.

Following targeted inactivation by homologous recombination in embryonic stem cells (Fig. 1A), intercrosses of *Adamtsl2*^+/−^ mice produced *Adamtsl2*^−/−^ progeny in a Mendelian fraction (observed 27.8%; expected 25%) (supplementary material Table S1). Almost all *Adamtsl2*^−/−^ newborns were cyanotic, had gasping respiration and died within 24 h of birth. In contrast to wild-type (WT) lungs, those of newborn *Adamtsl2*^−/−^ mice sank in fixative, indicating poor aeration. The oropharyngeal tract of *Adamtsl2*^−/−^ mice was unaffected, their diaphragm was intact and the histology of intercostal muscle fibers showed normal structure, including peripheral localization of the nucleus, excluding myogenic or other non-pulmonary causes of respiratory insufficiency (data not shown). A few (3.1%) *Adamtsl2*^−/−^ mice survived for 1-2 weeks after birth and showed a dramatic reduction in size, along with tight skin and limb contractures.

Quantitative real-time PCR analysis of lungs (Fig. 1B) and western blotting with an anti-ADAMTSL2 polyclonal antibody (Fig. 1C) showed reduced RNA and protein, respectively. The ADAMTSL2 antibody specifically detected recombinant ADAMTSL2 (Fig. 1D) and did not cross-react with homologous thrombospondin type-1 repeats (TSRs) of ADAMTSL1 (also known as punctin-1) (Fig. 1E,F), indicating that the antibody is unlikely to recognize tandem TSR arrays found in other ADAMTSL proteins.

**Fig. 2.**
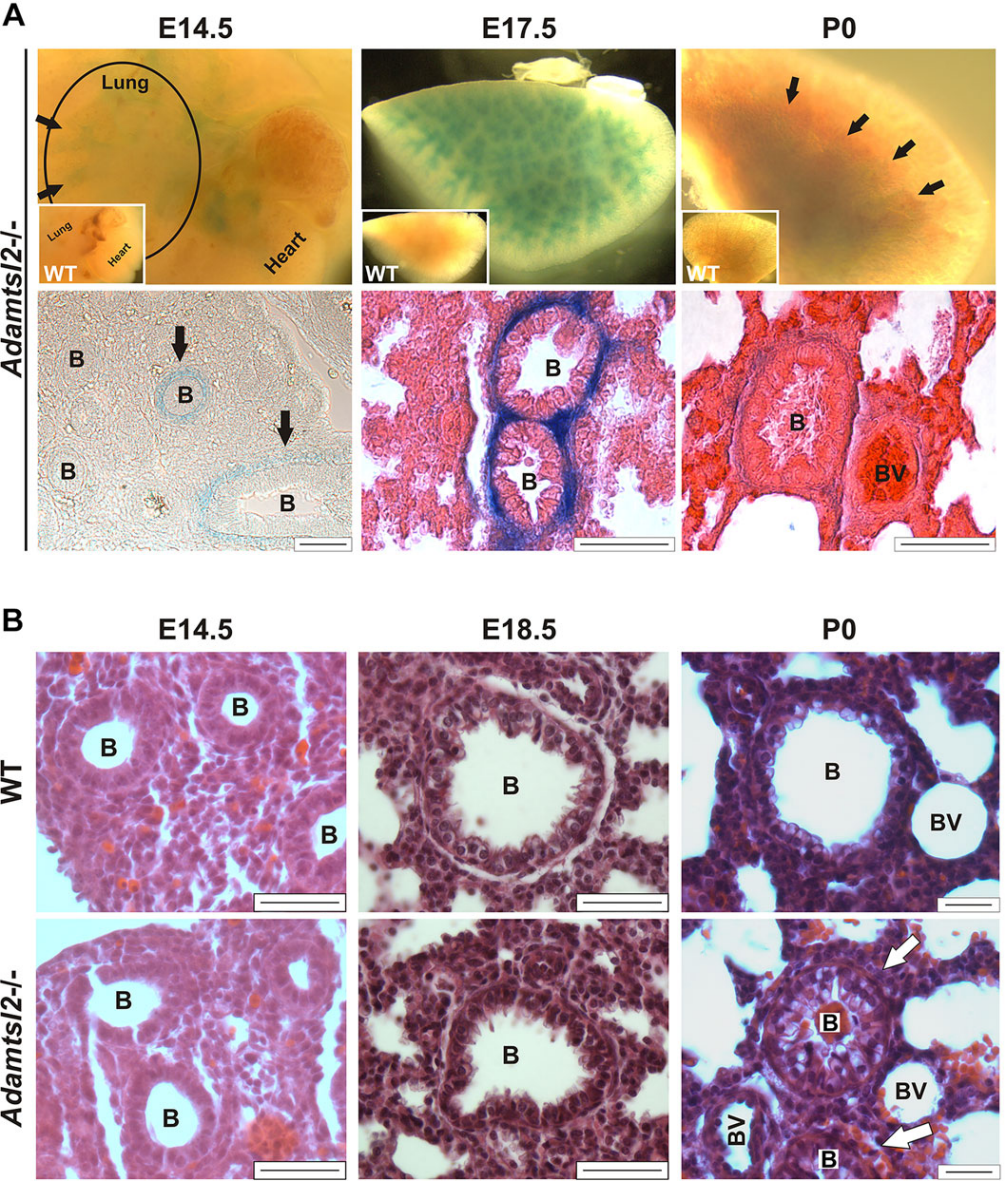
**Expression of *Adamtsl2* in lung and profound bronchial epithelial dysplasia in *Adamtsl2*^−/−^ mice.** (A) The top panels show representative β-galactosidase staining of whole lungs at embryonic day E14.5 (from *n*=3), E17.5 (from *n*=6) and postnatal day P0 (from *n*=3) (circle at E14.5 outlines the lung, with the heart still attached; arrows indicate stained bronchi; inset shows no β-galactosidase staining in WT tissue at E17.5). The lower panels show a β-galactosidase-stained section at E14.5 indicating emerging *Adamtsl2* expression in some bronchi (arrows), and eosin- and β-galactosidase-stained sections of lung tissue (center and right-hand panels) with strong expression of *Adamtsl2* around bronchi at E17.5 and reduced staining at P0. Scale bars: 50 µm. (B) Representative H&E staining of WT and *Adamtsl2*^−/−^ lung sections at E14.5 (from *n*>3), E18.5 (from *n*>5) and P0 (from *n*>6). Arrows show occluded bronchi at P0. *Adamtsl2*^−/−^ lungs at E14.5 and E18.5 showed minimal to no difference in bronchial development. Scale bars: 25 µm. B, bronchi; BV, blood vessel.

Whole-mount β-galactosidase staining of lungs, using the intragenic *lacZ* reporter, showed *Adamtsl2* gene expression exclusively in the smooth muscle cell (SMC) layer of the bronchial tree from embryonic day (E) 14.5 on to birth (Fig. 2A), but not elsewhere within the lung, consistent with prior *in situ* hybridization analysis (Koo et al., 2007). Staining intensity peaked at E17.5 and was reduced at birth. No β-galactosidase staining was seen in pulmonary parenchyma and vasculature of embryos. Postnatally, *Adamtsl2* expression was reduced further in bronchial SMCs and extended into the surrounding parenchyma (data not shown). Interestingly, in embryonic lungs from E14.5 to birth, we observed concurrent strong expression in some bronchi, yet weak expression in others within the same tissue sections, suggesting that even within the bronchial tree, *Adamtsl2* expression might be extremely dynamic.

Newborn *Adamtsl2*^−/−^ bronchial lumina were partially or completely occluded by a dysplastic bronchial epithelium, and the lumina contained vesicular material at birth (Fig. 2B, lower right). However, no comparable anomaly of the bronchial epithelium was obvious during embryonic development, although a few bronchi showed altered cross-sectional profiles (Fig. 2B, left, middle). Smooth muscle actin staining at P0 showed an intact, apparently unaltered, SMC layer and bronchial epithelial cells were all positive for cytokeratin, i.e. they retained epithelial differentiation (supplementary material Fig. S1A,B). Col IV staining indicated an intact and apparently normal bronchial epithelial basement membrane at birth (supplementary material Fig. S1F). Staining for markers of epithelial differentiation, PKCζ and ZO-2 indicated their presence in the epithelium as expected, but at reduced levels and with considerable spatial disorganization (supplementary material Fig. S1G,H,I). There was no significant alteration of cell proliferation or apoptosis within the bronchial wall (supplementary material Fig. S2).

**Fig. 3.**
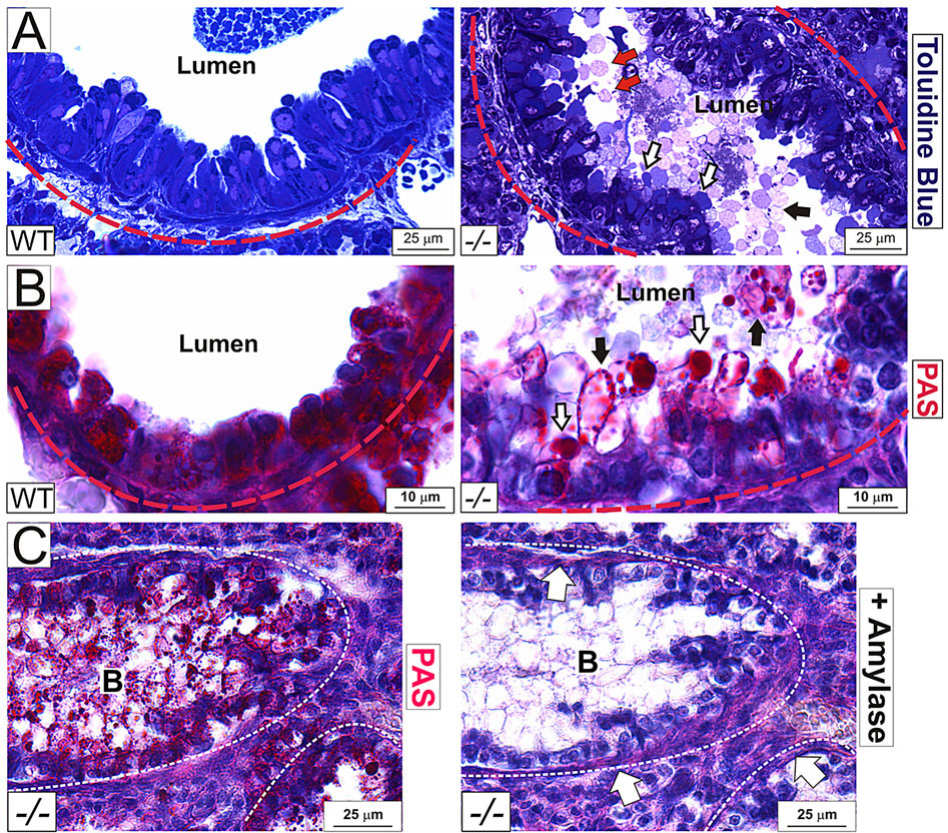
**Glycogen accumulation in the bronchial epithelium of *Adamtsl2*^−/−^ mice.** (A) Toluidine Blue staining of 1-µm-thick epoxy-embedded sections of lung tissue. The basement membrane is outlined by a red dashed line. Some vesicles occluding the lumen are stained strongly with Toluidine Blue (white arrows), whereas others, more centrally located, appeared unstained (black arrow). Vesicles attached to epithelial cells are marked with red arrows (from *n*=2 per genotype). (B,C) Vesicles in *Adamtsl2*^−/−^ mice contain glycogen as shown by periodic acid Schiff staining (B, PAS) which is sensitive to amylase digestion (C, right-hand panel). Note that the basement membrane PAS staining is amylase resistant (C, right-hand panel, white arrows). Some glycogen is contained within large structures (B, right-hand panel, white arrows), whereas some is found in smaller globules (B, right-hand panel, black arrows) (from *n*=4 per genotype). B, bronchus.

Newborn *Adamtsl2*^−/−^ bronchial epithelial cells were disorganized, with enlarged abnormal vesicles continuous with the epithelial apical surface (Fig. 3A). Both the intra-lumenal and epithelial vesicles stained strongly with periodic acid Schiff (PAS) stain (Fig. 3B), consistent with the previously documented appearance of GD tracheal, liver and cardiac biopsies, which had shown carbohydrate-enriched inclusions in these organs, and led to designation of GD as a glycoprotein storage disorder (Spranger et al., 1984; Shohat et al., 1990; Santolaya et al., 1997). We found that this PAS-positive material was sensitive to α-amylase digestion, indicative of substantial glycogen content (Fig. 3C). The vesicles stained positive for the endoplasmic reticulum (ER) marker KDEL, but did not stain with the lysosomal marker Lamp-2 or the Golgi marker GM-130 (supplementary material Fig. S1C-E), suggesting glycogen accumulation in the secretory pathway. Staining with Toluidine Blue did not result in metachromasia; Alcian Blue staining at acidic pH and biotinylation did not strongly label the vesicles, indicative of a lack of substantial proteoglycan, acid mucin or protein content, respectively (data not shown). Thus, although overall bronchial architecture was preserved, *Adamtsl2*^−/−^ newborn mice had severe bronchial epithelial dysplasia.

**Fig. 4.**
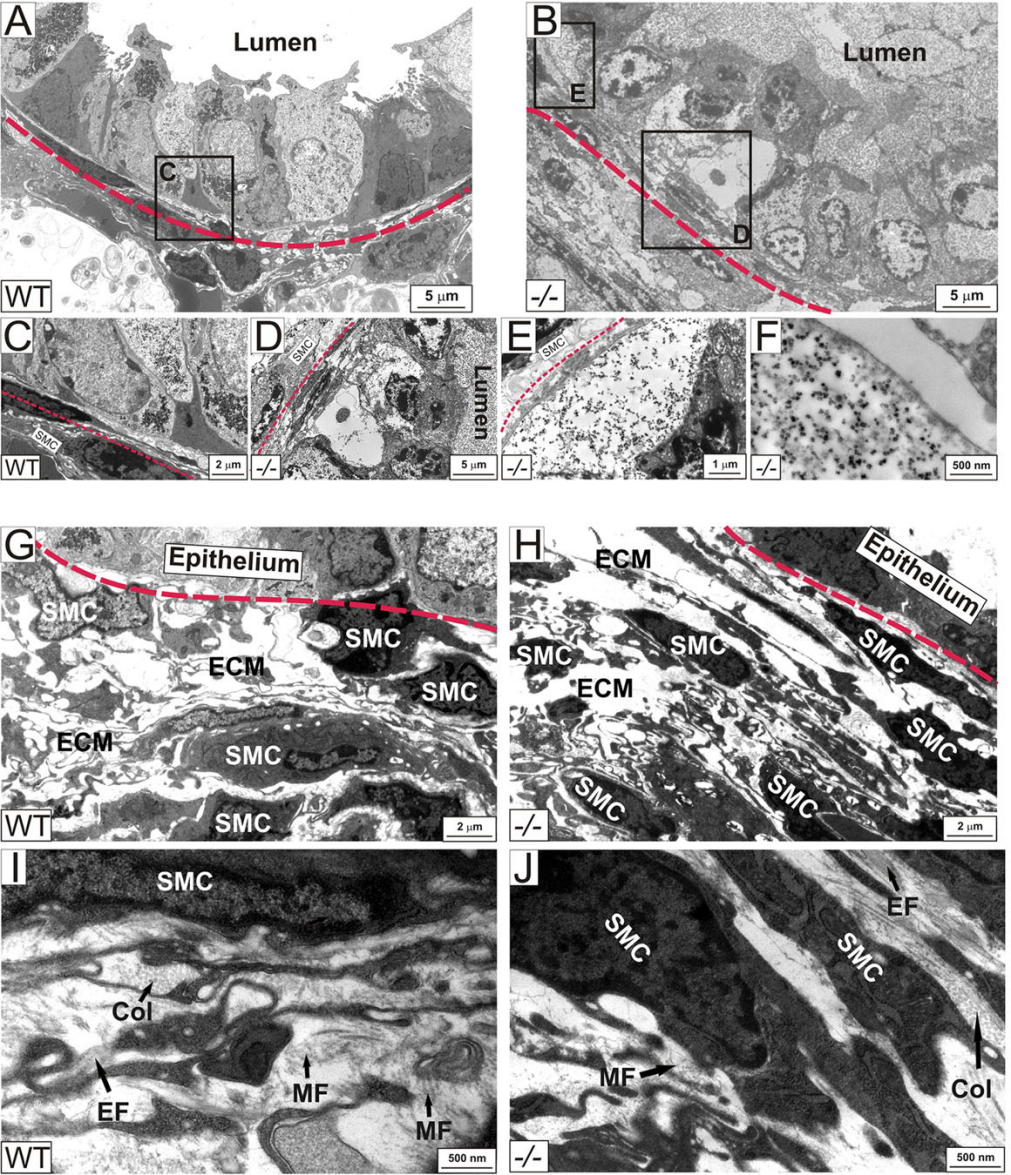
**Ultrastructure of the bronchial wall of *Adamtsl2*^−/−^ mice.** (A-F) Ultrastructural analysis of the newborn bronchial wall using TEM. In contrast to WT bronchial epithelium (A,C), the bronchial epithelium in *Adamtsl2*^−/−^ (−/−) mice is disorganized (B,D,E) and membrane-bound vesicles engorged with granular, electron dense material fill mutant cells and the lumen (E,F). (G-H) The SMC layer of the bronchial wall did not show consistent differences between WT and *Adamtsl2*^−/−^ mice. (I-J) Higher magnification of the ECM around the bronchial SMCs (from *n*=4 per genotype). B, Bronchi; Col, collagen fibers; EF, elastic fiber; EP, bronchial epithelium; MF, microfibril.

Electron microscopy of newborn lungs showed that *Adamtsl2*^−/−^ bronchial epithelial cells were engorged with membrane-bound vesicles containing a granular material similar to that found in the luminal vesicles (Fig. 4A,B). The intracellular vesicles were typically located apically, but were also found in some cells between the basement membrane and the nucleus (Fig. 4E). The vesicles contained a fine, granular, electron-dense material (Fig. 4F), with a similar appearance to that previously reported in GD biopsies of liver, skin, cartilage, and the heart valve (Spranger et al., 1984; Shohat et al., 1990; Pontz et al., 1996), but contained no intact organelles or visible chromatin. No consistent differences were detected in the morphology of the bronchial SMC layer (Fig. 4G,H). Thus, lack of ADAMTSL2 produced by bronchial SMCs led to a profound dysplasia of the bronchial epithelium at birth, but without apparently altering the bronchial SMC layer or overall lung morphology during the embryonic period.

### Dysregulation of ECM in the bronchial microenvironment characterizes *Adamtsl2*^−/−^ bronchi

**Fig. 5.**
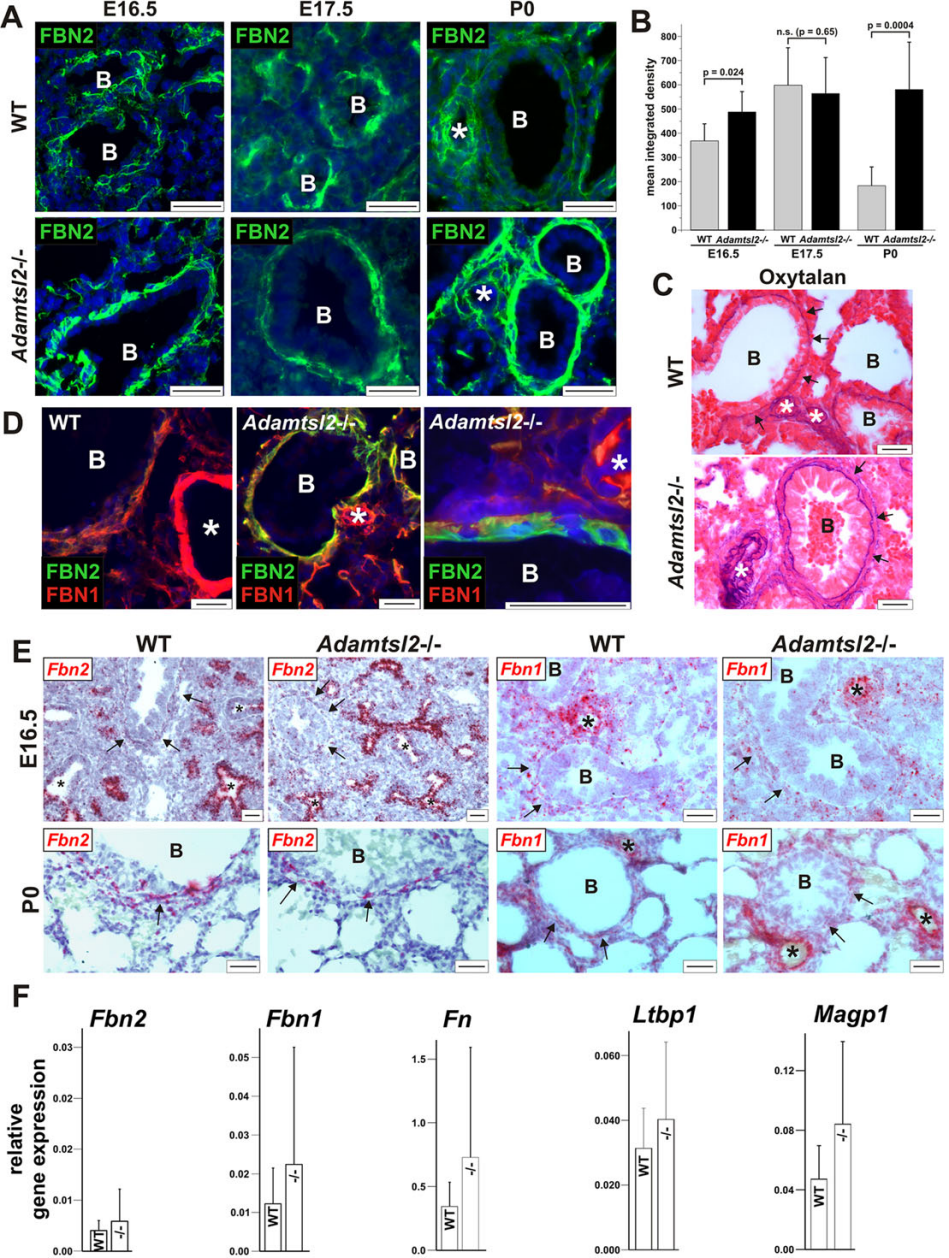
**Peribronchial microfibril dysregulation in *Adamtsl2*^−/−^ lungs.** (A) Immunolocalization of FBN2 (green) in frozen (E16.5) (from *n*=3 per genotype) or paraffin-embedded (E17.5, P0) (from *n*>6 per genotype) lung sections. Note the stronger FBN2 staining in *Adamtsl2*^−/−^ bronchi compared to the WT. The genotype of the E16.5 controls is *Adamtsl2*^+/−^. (B) Quantification of mean±s.d. integrated density of the fluorescence signal for FBN2 at E16.5 (*n*=3 per genotype), E17.5 (*n*=3 per genotype) and P0 (*n*>6 per genotype). A small, but statistically significant difference between the WT and *Adamtsl2*^−/−^ lungs was observed at E16.5. A strong increase in the mean integrated density was observed in the *Adamtsl2*^−/−^ lungs at P0. (C) The total amount of microfibrils is greater in the bronchial wall of newborn *Adamtsl2*^−/−^ lungs as assessed by histochemical staining for oxytalan fibers (arrows) (from *n*=4 per genotype). (D) Co-immunostaining of FBN2 (green) and FBN1 (red) in frozen sections from lung tissue at birth using antibodies covalently labeled with the respective fluorophores. Nuclei are counterstained with DAPI (blue). In the *Adamtsl2*^−/−^ bronchi, FBN2 staining predominates peri-bronchially, but is absent in blood vessels. In WT and mutant mice, FBN1 staining is present primarily in blood vessels (white asterisk), with weaker staining of the bronchial wall. The lower panel shows a magnified view of sub-epithelial FBN2 accumulation (green) in an *Adamtsl2*^−/−^ bronchus (from *n*=2 per genotype). (E) *In-situ* hybridization for *Fbn2* and *Fbn1* mRNA (red) shows that there is strong *Fbn2* expression in bronchial epithelium at E16.5 in WT and *Adamtsl2*^−/−^ mice (asterisks) (from *n*>4 per genotype). Note the absence of *Fbn2* expression in more developed bronchi (upper left panel) and weak *Fbn2* expression in the bronchial smooth muscle cell layer (arrows). *Fbn1* is predominantly expressed in the pulmonary blood vessels. (F) qRT-PCR analysis of expression of microfibril associated genes in lung at P0 normalized to expression of *Gapdh* (mean±s.d., *n*=3 per genotype). No significant changes were detected between *Adamtsl2*^−/−^ (^−/−^) and WT lungs. Scale bars: 25 µm. B, bronchi; asterisk, blood vessel.

The embryonic period is characterized by the expression of *Fbn2* rather than *Fbn1* mRNA (Mariencheck et al., 1995; Zhang et al., 1995). To determine whether ADAMTSL2 acted locally at the interface between the bronchial epithelium and the SMCs, we compared FBN2 and FBN1 staining around *Adamtsl2*^−/−^ and WT bronchi as well as in the adjacent vasculature, which does not express FBN2 or ADAMTSL2. We consistently found stronger FBN2 immunostaining in the bronchial SMC layer of *Adamtsl2*^−/−^ lungs at birth (P0) compared to WT (Fig. 5A,B). In contrast, FBN2 staining was unaltered in *Adamtsl2*^−/−^ bronchial arteries and in other tissues, where *Adamtsl2* is not expressed (Fig. 5A, asterisk; data not shown), suggesting that ADAMTSL2 acted locally in the bronchial ECM. Consistent with lower *Fbn1* mRNA expression until late gestation and postnatally, immunostaining showed weak FBN1 immunostaining at E14.5 around blood vessels in both *Adamtsl2*^−/−^ and WT mice (supplementary material Fig. S3). At E17.5 and at birth, staining for FBN1 microfibrils was strong around blood vessels, consistent with the localization of *Fbn1* gene expression, and did not differ between *Adamtsl2*^−/−^ and WT mice. At birth, FBN1 immunostaining seemed to be slightly enhanced or better defined at the interface between the bronchial epithelial and the SMCs in *Adamtsl2*^−/−^ bronchi. However, quantification of the FBN1 immunofluorescence signal did not result in statistically significant differences. The antibody-independent histochemical oxytalan stain to visualize all microfibrils regardless of fibrillin isoform composition (Inoue et al., 2012), showed consistently greater abundance of oxytalan fibers in *Adamtsl2*^−/−^ bronchi (Fig. 5C). In transmission electron microscopy (TEM) images, the microfibrils appeared to be structurally normal and we were not able to detect quantitative changes in microfibrils or other structural ECM components (Fig. 4I,J). This could be caused by the much higher magnification used to visualize microfibrils in TEM. Taken together, we concluded that the absence of ADAMTSL2 results in the persistence of fibrillin microfibrils in the bronchial wall at birth, but not in the adjacent vasculature, and that these microfibrils have a higher amount of FBN2 than WT bronchi.

To further resolve the spatial relationship between FBN1 and FBN2 microfibrils in the bronchial wall, we co-stained lung sections with antibodies against FBN1 and FBN2 that were directly labeled with fluorophores. As shown above, in newborn *Adamtsl2*^−/−^ mice, FBN2 staining was especially strong at the interface between the bronchial epithelium and SMCs, i.e. below the bronchial epithelium basement membrane, where it partially colocalized with FBN1 (Fig. 5D, center and right-hand panels). By using non-radioactive *in-situ* hybridization (ISH) for improved cellular resolution compared to the previously reported radioactive ISH (Mariencheck et al., 1995; Zhang et al., 1995), we found that *Fbn2* mRNA was localized to the bronchial epithelium of embryonic lungs, but was not detected in blood vessels (Fig. 5E). At E16.5, *Fbn2* mRNA was expressed at very low levels in the bronchial SMC layer as compared with the bronchial epithelium. However, bronchial SMC became the exclusive site of *Fbn2* expression at birth, although at much lower levels than observed in the embryonic bronchial epithelium prior to birth. *Fbn1* mRNA was strongly expressed in the blood vessels during embryonic development, but was detected only at low levels in the bronchial SMCs (Fig. 5E). The intensity of *Fbn1* or *Fbn2* mRNA expression observed by ISH was not different in *Adamtsl2*^−/−^ and WT lungs. We observed no changes in quantitative mRNA expression levels for *Fbn1* or *Fbn2* (Fig. 5F), suggesting that the observed staining differences between *Adamtsl2*^−/−^ and WT lungs results from post-transcriptional mechanisms, e.g. enhanced FBN2 assembly, altered intermolecular interactions, or reduced microfibril degradation or turnover.

**Fig. 6.**
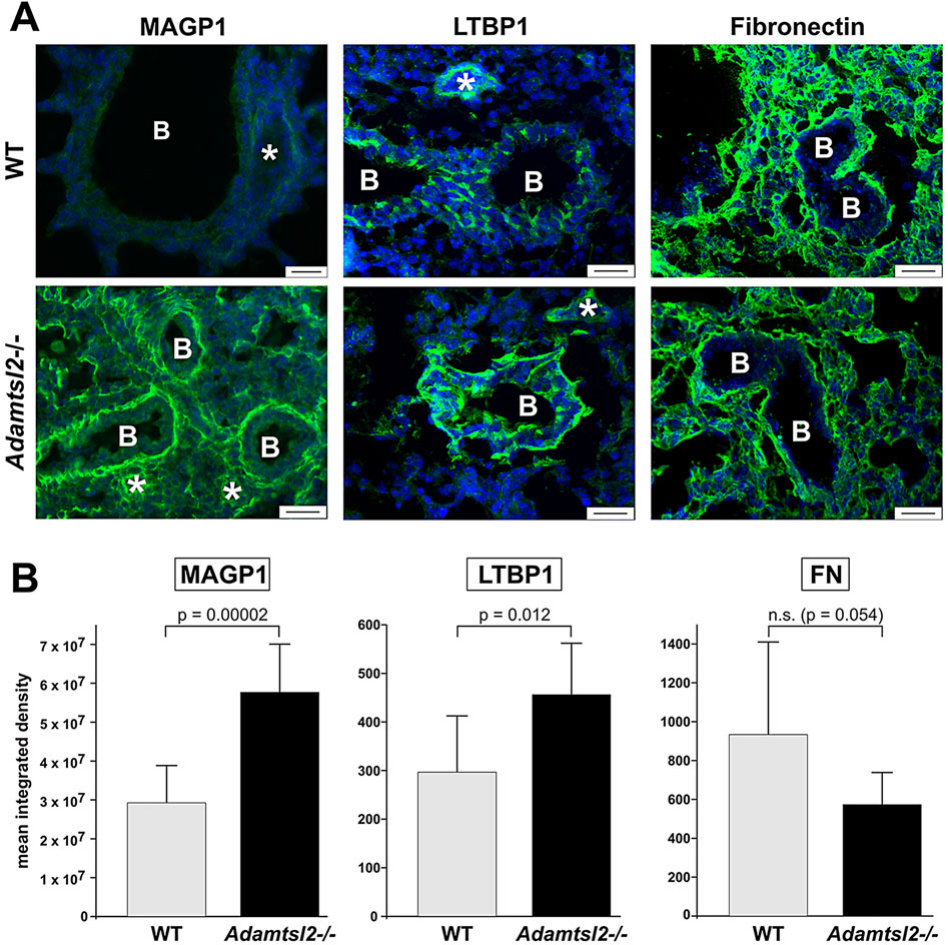
**Altered distribution of microfibril-associated ECM proteins in *Adamtsl2*^−/−^ lungs.** (A) Immunolocalization of the microfibril-associated proteins MAGP1, LTBP1 and fibronectin (FN) in frozen lung sections at P0. Note stronger MAGP1 and LTBP1 staining in bronchi and parenchyma or bronchial epithelium, respectively, in *Adamtsl2*^−/−^ lung (from *n*=4) compared to the WT (from *n*=3). Fibronectin appeared to be slightly reduced in *Adamtsl2*^−/−^ lungs compared to the WT. FN did not localize to the bronchial epithelium. (B) Quantification of mean±s.d. integrated density of fluorescence signals for the respective antibodies. A statistically significant increase in intensity was observed for MAGP1 and LTBP1 in *Adamtsl2*^−/−^ (*n*=4) lungs compared to the WT (*n*=3). Scale bars: 25 µm. B, bronchi; asterisk, blood vessel.

Staining for microfibril associated glycoprotein-1 (MAGP1), which localizes to microfibrils and binds to FBN1 and FBN2 *in vitro* (Segade et al., 2000, 2002), was strongly enhanced in bronchial SMC ECM of *Adamtsl2*^−/−^ mice at birth (Fig. 6A, left). Because ADAMTSL2, FBN1 and FBN2 interact with LTBP1 *in vitro* (Isogai et al., 2003; Le Goff et al., 2008), we compared the localization of LTBP1 in *Adamtsl2*^−/−^ and WT bronchi (Fig. 6A, middle). LTBP1 immunostaining was consistently stronger in *Adamtsl2*^−/−^ bronchial epithelium compared to the WT. However, in contrast to increased FBN2 and MAGP1 in *Adamtsl2*^−/−^ bronchi, LTBP1 was not localized to bronchial SMCs. Because fibronectin (FN) forms an ECM network necessary for the subsequent deposition of fibrillin microfibrils and LTBPs (Taipale et al., 1996; Kinsey et al., 2008; Sabatier et al., 2009; Zilberberg et al., 2012), we analyzed bronchial FN distribution in *Adamtsl2*^−/−^ and WT lungs (Fig. 6A, right). FN staining was comparable or slightly reduced in *Adamtsl2*^−/−^ lungs and its overall distribution was unaltered. Quantification showed a statistically significant increase in MAGP1 and LTBP1 in *Adamtsl2*^−/−^ lungs, whereas the levels of FN were unchanged (Fig. 6B). Therefore, the accumulation of FBN2 microfibrils cannot be explained by an augmentation of the FN scaffold. Gene expression levels of these ECM molecules in *Adamtsl2*^−/−^ and WT lung were comparable at birth (Fig. 5F).

### ADAMTSL2 directly binds to FBN2

**Fig. 7.**
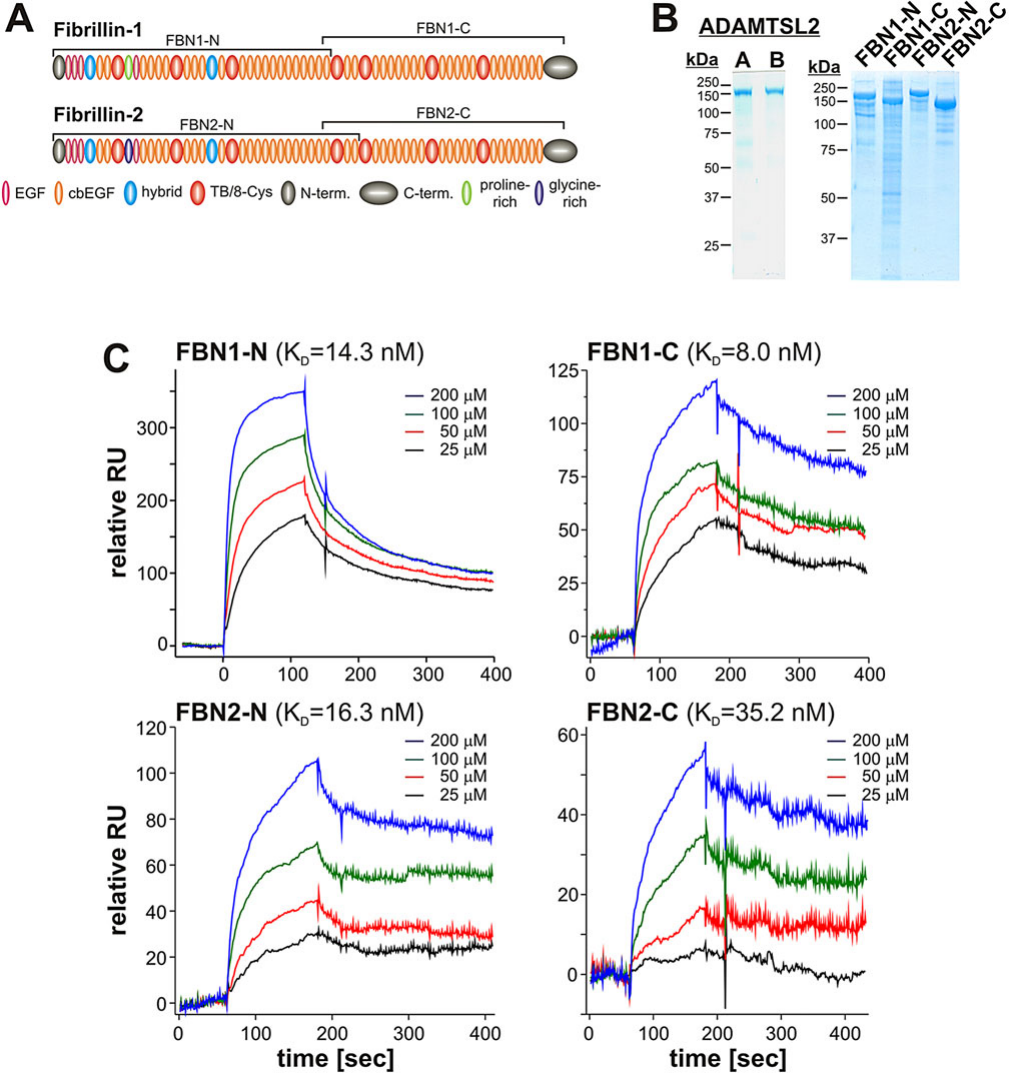
**ADAMTSL2 binds to FBN1 and FBN2.** (A) Domain structure of human FBN1 and FBN2. The peptides used in the binding assays with ADAMTSL2 are marked with brackets. (B) ADAMTSL2 (left, preparation A and B) and fibrillin peptides (right) were separated on 7.5% polyacrylamide gels under reducing conditions followed by Coomassie staining to demonstrate the purity of protein preparations. (C) Binding of immobilized ADAMTSL2 to soluble N- or C-terminal fragments of FBN1 or FBN2, respectively, was measured by surface plasmon resonance (Biacore) (*n*=2). The *K*_D_ for FBN1-N was similar to that previously published for a slightly different N-terminal fragment (60 nM) (Le Goff et al., 2011).

Because ADAMTSL2 is not a protease, nor is known to be functionally associated with proteases and therefore does not directly degrade FBN2, we asked whether it could bind to FBN2 as a way of influencing its assembly. ADAMTSL2 has previously been shown to interact with the N-terminal half of FBN1 (Le Goff et al., 2011). We used surface plasmon resonance technology to analyze the binding of recombinant ADAMTSL2 to recombinant halves of FBN2 and FBN1 (as a control) (Fig. 7A,B). We found that, in addition to binding to both halves of FBN1, ADAMTSL2 also bound to the N- and C-terminal halves of FBN2 with affinities that were comparable to those for FBN1 binding (Fig. 7C). Strongest binding of ADAMTSL2 was observed for the C-terminus of FBN1 (*K*_D_=8.0 nM) and weakest binding for the C-terminus of FBN2 (*K*_D_=35.2 nM). Interestingly, the N-terminal fragments of FBN1 and FBN2 showed very similar *K*_D_ values for their interaction with ADAMTSL2 (14.3 nM and 16.3 nM, respectively). These results indicate that there are at least two distinct high-affinity binding sites for ADAMTSL2 on both FBN1 and FBN2.

### Alterations in canonical TGFβ signaling precede bronchial epithelial dysplasia

**Fig. 8.**
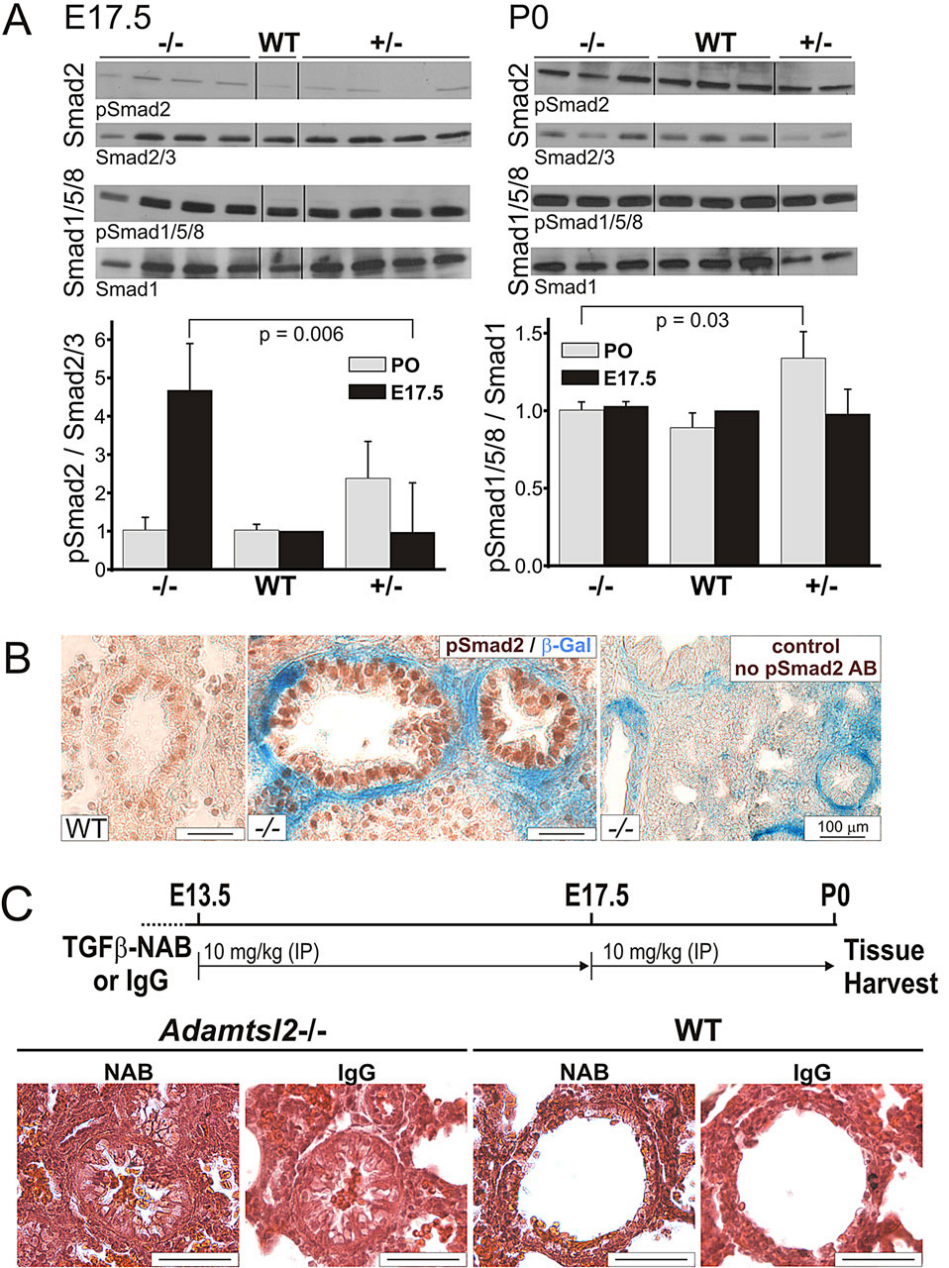
**Enhanced canonical TGFβ signaling in *Adamtsl2*^−/−^ bronchial epithelium.** (A) Western blots for pSmad2 (canonical TGFβ signaling) or pSmad1/5/8 (BMP signaling) and total Smad2/3 or Smad1, respectively, from lung protein extracts at E17.5 (left) and P0 (right) are shown (*n*=1 for WT, *n*=4 for *Adamtsl2*^−/−^; *n*=4 for *Adamtsl2*^+/−^) and P0 (right, *n*=3 for WT and *Adamtsl2*^−/−^; *n*=2 for *Adamtsl2*^+/−^). Equal amounts of protein were loaded and the mean±s.d. intensity per pSmad band was quantified using ImageJ and normalized to the intensity of the band for the respective total Smad (A, bottom) (*n*=3 for WT and *Adamtsl2*^−/−^; *n*=2 for *Adamtsl2*^+/−^). (B) pSmad2 immunohistochemistry shows strong nuclear staining in *Adamtsl2*^−/−^ bronchial epithelium (β-galactosidase staining for *Adamtsl2* expression in blue is in SMCs) at E17.5 compared to the WT (from *n*=3 per genotype). In the control section, the primary anti-pSmad2 antibody (AB) was omitted. (C) Experimental scheme for administration of pan-TGFβ neutralizing antibody (NAB) 1D11 and control IgG injection (top). H&E staining shows occluded bronchi in NAB-treated lungs from *Adamtsl2*^−/−^ mice (from *n*=7), but not from WT (from *n*=6). *Adamtsl2*^−/−^ (*n*=6) and WT (*n*=2) mice were analyzed from the IgG-injected control group. Scale bars: 25 µm, if not indicated otherwise.

Because of the observed changes in microfibril composition in *Adamtsl2*^−/−^ bronchi, and because fibrillins regulate and integrate TGFβ and BMP signaling (Neptune et al., 2003; Le Goff et al., 2008; Sengle et al., 2008; Nistala et al., 2010b; Holm et al., 2011), we analyzed the respective signaling pathways (Fig. 8; supplementary material Fig. S4). Smad2 phosphorylation was increased in *Adamtsl2*^−/−^ lung extracts at E17.5, but not at birth (Fig. 8A), with strong nuclear staining for phosphorylated SMAD2 (pSmad2) in bronchial epithelial cells of E17.5 *Adamtsl2*^−/−^ mice (Fig. 8B), indicative of enhanced canonical TGFβ signaling. There were no consistent staining differences for pSmad2 in WT versus *Adamtsl2*^−/−^ SMCs or parenchyma (Fig. 8B, compare left and middle panel). BMP signaling (as assessed by measuring the phosphorylation of Smad1/5/8) (Fig. 8A) and non-canonical TGFβ signaling pathways (as assessed by measuring phosphorylation of Erk1/2 or p38) were comparable in WT and *Adamtsl2*^−/−^ lungs (supplementary material Fig. S4A). We injected TGFβ-neutralizing antibody (NAB) at 13.5 and 17.5 days of gestation into pregnant *Adamtsl2*^+/−^ dams crossed with *Adamtsl2*^+/−^ males, and analyzed the lungs of newborn mice (Fig. 8C). Although absence of nuclear pSmad2 staining in both wild-type and null lungs showed the effectiveness of the treatment with TGFβ neutralizing antibody (supplementary material Fig. S4B), this treatment did not rescue bronchial epithelial dysplasia in *Adamtsl2*^−/−^ lungs when compared with the IgG control (Fig. 8C).

## DISCUSSION

We have discovered that, in contrast to GD and MLS, the absence of ADAMTSL2 in mice manifests as neonatal death, probably resulting from bronchial occlusion. This finding is relevant to GD given that narrowing of the trachea and major bronchi, as well as recurrent respiratory infections, are observed in children with GD (Shohat et al., 1990; Panagopoulos et al., 2005; Giray et al., 2008). To be detrimental, bronchial narrowing would not need to involve the entire length of the airways because local occlusion of proximal airways would result in the lack of aeration distally. Indeed, *Adamtsl2* expression in bronchi is dynamic, and the observed bronchial abnormalities were not equally severe in all bronchi. A small ventricular septal defect was also present in these mice and will be described elsewhere.

Previous ultrastructural analysis of GD liver, cartilage and skin biopsies has identified large membrane-bound cellular vesicles containing a fine granular material (Spranger et al., 1984; Shohat et al., 1990; Santolaya et al., 1997). However, the identity of the material was not known. Here, in *Adamtsl2*^−/−^ bronchi, we unequivocally identified this material as glycogen for the first time, but the mechanism underlying its accumulation remains unclear. Although we observed bronchial epithelial TGFβ dysregulation at E17.5, this did not persist to birth, and treatment with TGFβ-neutralizing antibody during mid-to-late gestation did not remedy bronchial epithelial dysplasia or glycogen accumulation in *Adamtsl2*^−/−^ lungs.

Analysis of bronchial ECM provided additional new and unexpected observations. We observed that *Adamtsl2*^−/−^ bronchi at birth had more microfibrils, consisting mainly of FBN2, as well as increased associated MAGP1, whereas the morphology of SMCs, the cells that express *Adamtsl2* mRNA, was unaltered as assessed by light microscopy and TEM. FBN2 constitutes the major fibrillin isoform during the embryonic period. As shown here and reported previously, FBN2 is expressed and secreted by bronchial epithelial cells but is present only around bronchial SMCs, as shown by immunofluorescence microscopy (Zhang et al., 1995). The switch from FBN2 as the prevalent isoform, to FBN1 occurs around birth, when *Fbn2* transcription decreases while *Fbn1* mRNA is increased (Mariencheck et al., 1995; Zhang et al., 1995). Analysis of microfibril assembly in cultured fibroblasts has shown that in the presence of FBN1 and FBN2, both are assembled into microfibrils without apparent bias for either isoform (Beene et al., 2013). Enhancement of FBN1 assembly and/or binding to recombinant fibrillin fragments has been shown for other members of the ADAMTS family, including ADAMTS10, ADAMTSL4 and ADAMTSL6 (Kutz et al., 2011; Saito et al., 2011; Gabriel et al., 2012). Given that FBN2 accumulates around bronchial SMCs in the absence of ADAMTSL2, it is possible that ADAMTSL2 regulates FBN2 assimilation into microfibrils during the embryonic period, such as by modulating the avidity for FBN2 binding to FN or FBN2 self-assembly. Previous studies have identified FN and heparan-sulfate-containing proteoglycans as requirements for microfibril assembly because their absence abolishes the formation of microfibrils (Tiedemann et al., 2001; Kinsey et al., 2008; Sabatier et al., 2009; Baldwin et al., 2014). ADAMTSL2 offers a potentially new mechanism that acts locally to selectively restrict FBN2 incorporation into microfibrils in a tissue-specific manner. ADAMTSL2 bound to FBN1 and FBN2 with similar affinities, and it is possible that ADAMTSL2 could similarly control FBN1 assembly into microfibrils. However, owing to perinatal lethality, a similar effect on FBN1 could not be addressed in this mouse model. Moreover, *Adamtsl2* expression seemed to be downregulated in the bronchi after E17.5, suggesting it could be a mechanism restricted to the embryonic period. Alternatively, ADAMTSL2 could perform isoform-specific functions, for which one would predict isoform-specific binding sites for ADAMTSL2 on FBN1 or FBN2. In this regard, future studies are required to map the binding sites of FBN1 and FBN2 on ADAMTSL2.

The finding that ADAMTSL2 is expressed in bronchial SMCs and that microfibrils formed subjacent to the bronchial basement membrane influence the integrity of bronchial epithelial cells, suggests that the cells might respond specifically to the composition and/or quantity of microfibrils. FBN2 microfibrils are structurally different from FBN1 microfibrils (Carta et al., 2006). FBN2 harbors two RGD sites (one RGD site in FBN1) and contains a distinctive glycine-rich domain (whereas there is a proline-rich domain in FBN1) (Piha-Gossack et al., 2012). The RGD sites on FBN1 and FBN2 differentially influence cell behavior of pulmonary fibroblasts (Sakamoto et al., 1996; McGowan et al., 2008). However, a direct effect on epithelial cells is unlikely, because a basement membrane separates bronchial epithelial cells from bronchial SMC. Alternatively, the observed phenotypic changes in the bronchial epithelium could be caused by microfibril-associated proteins, including growth factors, whose localization and quantity in the cellular microenvironment would depend on the specific subtype of microfibrils formed. For example, in the tight skin mouse, mutant FBN1 microfibrils enhance MAGP2 and collagen I deposition in the skin (Lemaire et al., 2004). FBN2 directly interacts with MAGP1 and MAGP2, and MAGP1 inhibits LTBP1 binding to fibrillin microfibrils, resulting in elevated TGFβ signaling (Penner et al., 2002; Werneck et al., 2004; Massam-Wu et al., 2010). We showed an increase of LTBP1 deposition in bronchial epithelial cells, concomitant with an excess of FBN2 microfibrils and MAGP1 in mutant bronchial SMC. Finally, the dysregulation of soluble growth factors tethered to these ECM complexes could be responsible for the alterations in the bronchial epithelium. Although we observed TGFβ dysregulation in *Adamtsl2*^−/−^ bronchial epithelium during late development, it was normal at birth and TGFβ-neutralizing antibody treatment did not reverse the abnormal bronchial epithelial morphology or glycogen accumulation. The neutralizing antibody used in this study has previously been shown to efficiently reduce TGFβ signaling during embryonic development when employed at a similar dose range that used here (Neptune et al., 2003; Nakanishi et al., 2007). Therefore, we conclude that a different mechanism, not involving TGFβ must be invoked to explain the bronchial epithelial anomaly.

That mutations in ADAMTSL2 cause different phenotypes in humans, dogs and mice suggests potential modifier genes in the respective species. GD and MLS share stiff or tight skin, joint contractures and short stature. GD and the novel mouse model of ADAMTSL2 deficiency presented here, share pulmonary and cardiac involvement with GD. The ventricular septal defect is a unique feature of the mouse model that is not commonly seen in GD, which usually results in postnatal aortic valve thickening. However, the bronchial epithelial dysplasia seen in *Adamtsl2*^−/−^ mice authentically replicates a specific effect of *Adamtsl2* deficiency on target cells, which we have characterized here in detail, identifying glycogen as a major component of the enlarged vesicles.

In summary, the present work provides a new mouse model of GD and identifies a previously unsuspected binding of ADAMTSL2 to FBN2, and effect on FBN2 assembly. We further demonstrate that the bronchial occlusion is not a consequence of TGFβ dysregulation. Future studies will address the mechanisms by which ADAMTSL2 regulates FBN2 microfibrils. Although global *Adamtsl2* inactivation leads to neonatal death, tissue specific conditional deletion, for which this *Adamtsl2*^−/−^ allele can be used, could provide insights on effects of ADAMTSL2 deficiency in the limbs and skin.

## MATERIALS AND METHODS

### Transgenic mice

C57BL/6N mice with a targeted deletion of *Adamtsl2* were obtained from the KOMP initiative (NIH, Bethesda, MD). They were crossed to C57BL/6J-TgN (*Zp3-Cre*)93Knw mice (Jackson Laboratory, Bar Harbor, ME) to delete the *Neo* cassette (Fig. 1A). *Neo-*deleted mice were subsequently used for β-galactosidase staining, thus utilizing intragenic *lacZ* as a surrogate for *Adamtsl2* mRNA expression. All mice were maintained in the C57BL/6N genetic background at the Biological Resources Unit of the Cleveland Clinic under protocols approved by the Institutional Animal Care and Use Committee (protocol numbers 2011-0557 and 2012-0818) with a 12-h-light–12-h-dark cycle, controlled temperature and food and water *ad libitum*.

### Genotyping and tissue harvesting

Genomic DNA isolated from tail biopsies was used for genotyping by PCR with specific forward and reverse primers (supplementary material Table S2). Primers were purchased from Integrated DNA Technology (Coralville, IA). *Adamtsl2*^−/−^ mice and wild-type littermates (used as controls in all aspects of this work) were obtained from *Adamtsl2*^+/−^ intercrosses. Mouse tissues were obtained after euthanasia was performed as recommended by the American Veterinary Medical Association Panel on Euthanasia.

### β-Galactosidase staining

Tissue was fixed in 4% paraformaldehyde (Electron Microscopy Sciences, Hatfield, PA) overnight and stained with potassium ferrocyanide, potassium ferricyanide and 5-bromo-4-chloro-3-indolyl-β-D-galactopyranoside (X-gal) (Denville Scientific, South Plainfield, NJ) as described previously (McCulloch et al., 2009).

### Histology and immunostaining

Tissue was fixed in 4% paraformaldehyde in phosphate-buffered saline (PBS, pH 7.2). Paraffin sections were used for hematoxylin & eosin, periodic-acid Schiff (PAS) and Masson trichrome staining according to standard procedures. PAS staining was performed with or without pretreatment with human amylase from saliva for 30 min at room temperature. Elastic fibers were visualized using the Hart's stain. The antibodies and antigen retrieval steps used for immunofluorescence and immunohistochemistry are summarized in supplementary material Table S3. The polyclonal fibrillin-1 (anti-rF6H), fibrillin-2, MAGP1 and LTBP1-K antibodies were as described previously (Tiedemann et al., 2001; Trask et al., 2001; Chen et al., 2007; Weinbaum et al., 2008). Fluorophore-conjugated secondary antibodies were purchased from Life Technologies (Grand Island, NY). TUNEL staining for apoptotic cells was performed using the DeadEnd Fluorometric TUNEL System (Promega, Madison, WI) following the manufacturer's instructions. To quantify the fluorescence signal, the mean intensity of the respective single channel from 2-4 frames of three biological replicates was measured using ImageJ (NIH) and mean value and standard deviation were calculated using the Origin software package (Originlab). Experimental and control groups were compared using the two-tailed Student's *t*-test and *P*<0.05 was considered statistically significant.

### Staining for oxytalan fibers

De-paraffinized sections were incubated for 30 min in 10% Oxone (catalog number 208036, Sigma, St Louis, MO), rinsed with water and 70% ethanol, stained for 1 h in aldehyde-fuchsin (pH 1) (#26328-01, Electron Microscopy Sciences, Hatfield, PA), rinsed in 95% ethanol and counterstained with eosin (Inoue et al., 2012).

### Biotinylation of tissue sections

To biotinylate lung tissue, sections were incubated for 2 min in PBS (pH 8.0) followed by 30 min with 10 mM Sulfo-NHS-LC-Biotin (Thermo Fisher Scientific, Waltham, MA) in PBS (pH 8.0). Sections were washed twice in distilled water and once with 0.1 M sodium carbonate. Subsequently, sections were blocked with 5% normal goat serum in PBS (pH 7.2) for 30 min at room temperature and incubated with a streptavidin–rhodamine conjugate (Jackson ImmunoResearch Laboratories, West Grove, PA) (1:200 in blocking buffer) for 1 h at room temperature. After washing, slides were mounted with ProLong Gold/DAPI (Life Technologies, Grand Island, NY).

### Electron microscopy

Lungs were fixed with 2.5% glutaraldehyde with 4% paraformaldehyde in 0.2 M sodium cacodylate buffer, pH 7.4, and embedded in epoxy resin. Thin sections (85 nm) were stained with osmium tetroxide and viewed with a Phillips CM12/STEM transmission electron microscope (FEI Company, Delmont, PA) equipped with a digital 11-megapixel CCD camera (Gatan, Pleasanton, CA).

### Quantitative real-time PCR

Total RNA was extracted from freshly dissected lung tissue using TRIzol Reagent (Life Technologies, Grand Island, NY), according to the manufacturer's protocol. The concentration and purity of the RNA was determined with the Nanodrop ND-1000 spectrophotometer (Thermo Fisher Scientific, Waltham, MA). Reverse transcription of 1 µg total RNA was performed using the High Capacity cDNA Reverse Transcription Kit (Life Technologies, Grand Island, NY). Quantitative real-time PCR (qRT-PCR) was performed in triplicates in 384-well hard-shell PCR plates (Bio-Rad, Hercules, CA) using 0.125 µl cDNA and SybrGreen master mix (Life Technologies, Grand Island, NY) in a total volume of 10 µl in a CFX384 Touch Real-Time PCR Detection System (Bio-Rad, Hercules, CA). Primers were designed using the PrimerQuest Design Tool (Integrated DNA Technologies, Coralville, IA) and purchased from the same supplier. The sequences of the primers are listed in supplementary material Table S2. PCR conditions were: (1) 50°C, 2 min; (2) 95°C, 10 min; (3) 95°C, 15 s; (4) 60°C, 1 min, (5) repeat step 3 and 4 for 39 cycles. ΔCT values were normalized to those for *Gapdh* or *Hprt1*.

### *In-situ* hybridization

Sections (6-µm thick) were de-paraffinized and hybridized with a specific probe for mouse *Fbn2* or *Fbn1* mRNA (Advanced Cell Diagnostics, Hayward, CA). Hybridization and detection was performed using the RNAScope 2.0 HD Red detection kit and HybEZ™ oven (Advanced Cell Diagnostics, Hayward, CA) according to the manufacturer's instructions.

### Western blotting and blot quantification

Lung protein extracts were prepared by homogenizing lung tissue (Ultra-Turrax T25) in 20 ml/g (v/w) T-PER Reagent (Thermo Fisher Scientific, Waltham, MA) including protease inhibitor (Complete Mini EDTA-free, Roche, Indianapolis, IN) and phosphatase inhibitor (PhosStop, Roche, Indianapolis, IN). After homogenization, samples were ultrasonicated (QSonic) and cleared (20,000 ***g***, 5-10 min, 4°C). 100–200 µg of protein per lane were separated under reducing conditions by SDS-PAGE, and western blotting was performed as described previously (Kutz et al., 2011). Cloning and purification of ADAMTSL1 (punctin-1) and ADAMTSL2 were as described previously (Hirohata et al., 2002; Koo et al., 2007). Primary antibodies are listed in supplementary material Table S3. For quantification, blots were scanned, the integral density of specific bands was measured using ImageJ, and the ratio of phosphorylated to total protein was used for statistical analysis using the two-tailed Student’s *t*-test (OriginLab software).

### Intermolecular interaction analysis by surface plasmon resonance

Purified fibrillin peptides and ADAMTSL2 were diluted in 10 mM MES buffer, pH 6.0 and immobilized on a BIAcore CM5 sensor chip (research grade, GE Healthcare, Piscataway, NJ) with the amine coupling kit according to the manufacturer's instructions. About 1500 resonance units were coupled to the chip for analysis in a BIAcore 3000 instrument (GE Healthcare, Piscataway, NJ). The kinetic analysis was performed at 25°C in 10 mM HEPES buffer, pH 7.4 including 150 mM NaCl, 2 mM CaCl_2_ and 0.005% (v/v) surfactant P20 (running buffer) at a flow rate of 20 μl/min. Purified recombinant fibrillin-1 and -2 fragments, as described previously (Jensen et al., 2001; Lin et al., 2002), were diluted in running buffer at different concentrations and injected through an uncoupled control flow cell and the flow cell coupled to ADAMTSL2 (Koo et al., 2007). Association was allowed for 3-6 min followed by a 10-min dissociation phase. 1 M NaCl with 2–10% (v/v) acetonitrile was used for regeneration after each injection at a flow rate of 50 μl/min for 30-60 s. The stabilization time following the regeneration was 2 min.

### TGFβ neutralizing antibody treatment

*Adamtsl2*^+/−^ dams from timed pregnancies after crosses to *Adamtsl2*^+/−^ males were injected intraperitoneally at E13.5 and E17.5 with the pan-TGFβ-neutralizing antibody 1D11 or an isotype matched IgG control (10 mg/kg body weight in PBS), both purchased from R&D Systems (Minneapolis, MN). Lung tissue was harvested at P0 and processed for histology as described above. Intraperitoneal injection of this antibody is commonly used to neutralize active TGFβ in mouse tissue *in vivo* (Neptune et al., 2003; Nakanishi et al., 2007). Moreover, it has been shown that specifically the IgG1 antibodies subtype, to which the TGFβ 1D11 antibody belongs, can cross the placental barrier (Paoletti et al., 2000; Pentsuk and van der Laan, 2009; Qi et al., 2012).

## Supplementary Material

Supplementary Material

## References

[DMM017046C1] Allali S., Le Goff C., Pressac-Diebold I., Pfennig G., Mahaut C., Dagoneau N., Alanay Y., Brady A. F., Crow Y. J., Devriendt K. et al. (2011). Molecular screening of ADAMTSL2 gene in 33 patients reveals the genetic heterogeneity of geleophysic dysplasia. *J. Med. Genet.* 48, 417-421 10.1136/jmg.2010.087544 21415077 PMC4413937

[DMM017046C2] Arteaga-Solis E., Gayraud B., Lee S. Y., Shum L., Sakai L. and Ramirez F. (2001). Regulation of limb patterning by extracellular microfibrils. *J. Cell Biol.* 154, 275-282 10.1083/jcb.200105046 11470817 PMC2150751

[DMM017046C3] Bader H. L., Ruhe A. L., Wang L. W., Wong A. K., Walsh K. F., Packer R. A., Mitelman J., Robertson K. R., O'Brien D. P., Broman K. W. et al. (2010). An ADAMTSL2 founder mutation causes Musladin-Lueke Syndrome, a heritable disorder of beagle dogs, featuring stiff skin and joint contractures. *PLoS ONE* 5, e12817 10.1371/journal.pone.0012817 20862248 PMC2941456

[DMM017046C4] Baldwin A. K., Cain S. A., Lennon R., Godwin A., Merry C. L. R. and Kielty C. M. (2014). Epithelial-mesenchymal status influences how cells deposit fibrillin microfibrils. *J. Cell Sci.* 127, 158-171 10.1242/jcs.134270 24190885 PMC3874785

[DMM017046C5] Beene L. C., Wang L. W., Hubmacher D., Keene D. R., Reinhardt D. P., Annis D. S., Mosher D. F., Mecham R. P., Traboulsi E. I. and Apte S. S. (2013). Nonselective assembly of fibrillin 1 and fibrillin 2 in the rodent ocular zonule and in cultured cells: implications for marfan syndrome. *Invest. Ophthalmol. Vis. Sci.* 54, 8337-8344 10.1167/iovs.13-13121 24265020 PMC3875392

[DMM017046C6] Brooke B. S., Habashi J. P., Judge D. P., Patel N., Loeys B. and Dietz H. C. III (2008). Angiotensin II blockade and aortic-root dilation in Marfan's syndrome. *N. Engl. J. Med.* 358, 2787-2795 10.1056/NEJMoa0706585 18579813 PMC2692965

[DMM017046C7] Carta L., Pereira L., Arteaga-Solis E., Lee-Arteaga S. Y., Lenart B., Starcher B., Merkel C. A., Sukoyan M., Kerkis A., Hazeki N. et al. (2006). Fibrillins 1 and 2 perform partially overlapping functions during aortic development. *J. Biol. Chem.* 281, 8016-8023 10.1074/jbc.M511599200 16407178 PMC3052983

[DMM017046C8] Chen Q., Sivakumar P., Barley C., Peters D. M., Gomes R. R., Farach-Carson M. C. and Dallas S. L. (2007). Potential role for heparan sulfate proteoglycans in regulation of transforming growth factor-beta (TGF-beta) by modulating assembly of latent TGF-beta-binding protein-1. *J. Biol. Chem.* 282, 26418-26430 10.1074/jbc.M703341200 17580303

[DMM017046C9] Cohn R. D., van Erp C., Habashi J. P., Soleimani A. A., Klein E. C., Lisi M. T., Gamradt M., ap Rhys C. M., Holm T. M., Loeys B. L. et al. (2007). Angiotensin II type 1 receptor blockade attenuates TGF-beta-induced failure of muscle regeneration in multiple myopathic states. *Nat. Med.* 13, 204-210 10.1038/nm1536 17237794 PMC3138130

[DMM017046C10] Cook J. R., Carta L., Benard L., Chemaly E. R., Chiu E., Rao S. K., Hampton T. G., Yurchenco P., Gen T. A. C. R. C., Costa K. D. et al. (2014). Abnormal muscle mechanosignaling triggers cardiomyopathy in mice with Marfan syndrome. *J. Clin. Invest.* 124, 1329-1339. 24531548 10.1172/JCI71059 PMC3934180

[DMM017046C11] Corson G. M., Charbonneau N. L., Keene D. R. and Sakai L. Y. (2004). Differential expression of fibrillin-3 adds to microfibril variety in human and avian, but not rodent, connective tissues. *Genomics* 83, 461-472 10.1016/j.ygeno.2003.08.023 14962672

[DMM017046C12] Doyle J. J., Gerber E. E. and Dietz H. C. (2012). Matrix-dependent perturbation of TGFbeta signaling and disease. *FEBS Lett.* 586, 2003-2015 10.1016/j.febslet.2012.05.027 22641039 PMC3426037

[DMM017046C13] Gabriel L. A. R., Wang L. W., Bader H., Ho J. C., Majors A. K., Hollyfield J. G., Traboulsi E. I. and Apte S. S. (2012). ADAMTSL4, a secreted glycoprotein widely distributed in the eye, binds fibrillin-1 microfibrils and accelerates microfibril biogenesis. *Invest. Ophthalmol. Vis. Sci.* 53, 461-469 10.1167/iovs.10-5955 21989719 PMC3292378

[DMM017046C14] Giray Ö., Kýr M., Bora E., Saylam G., Ugurlu B. and Gürel D. (2008). Clinical and morphological phenotype of geleophysic dysplasia. *Ann. Trop. Paediatr.* 28, 161-164 10.1179/146532808X302206 18510828

[DMM017046C15] Habashi J. P., Judge D. P., Holm T. M., Cohn R. D., Loeys B. L., Cooper T. K., Myers L., Klein E. C., Liu G., Calvi C. et al. (2006). Losartan, an AT1 antagonist, prevents aortic aneurysm in a mouse model of Marfan syndrome. *Science* 312, 117-121 10.1126/science.1124287 16601194 PMC1482474

[DMM017046C16] Habashi J. P., Doyle J. J., Holm T. M., Aziz H., Schoenhoff F., Bedja D., Chen Y., Modiri A. N., Judge D. P. and Dietz H. C. (2011). Angiotensin II type 2 receptor signaling attenuates aortic aneurysm in mice through ERK antagonism. *Science* 332, 361-365 10.1126/science.1192152 21493863 PMC3097422

[DMM017046C17] Hirohata S., Wang L. W., Miyagi M., Yan L., Seldin M. F., Keene D. R., Crabb J. W. and Apte S. S. (2002). Punctin, a novel ADAMTS-like molecule (ADAMTSL-1) in extracellular matrix. *J. Biol. Chem.* 22, 22. 10.1074/jbc.M109665200 11805097

[DMM017046C18] Holm T. M., Habashi J. P., Doyle J. J., Bedja D., Chen Y., van Erp C., Lindsay M. E., Kim D., Schoenhoff F., Cohn R. D. et al. (2011). Noncanonical TGFbeta signaling contributes to aortic aneurysm progression in Marfan syndrome mice. *Science* 332, 358-361 10.1126/science.1192149 21493862 PMC3111087

[DMM017046C19] Inoue K., Hara Y. and Sato T. (2012). Development of the oxytalan fiber system in the rat molar periodontal ligament evaluated by light- and electron-microscopic analyses. *Ann. Anat.* 194, 482-488 10.1016/j.aanat.2012.03.010 22727934

[DMM017046C20] Isogai Z., Ono R. N., Ushiro S., Keene D. R., Chen Y., Mazzieri R., Charbonneau N. L., Reinhardt D. P., Rifkin D. B. and Sakai L. Y. (2003). Latent transforming growth factor beta-binding protein 1 interacts with fibrillin and is a microfibril-associated protein. *J. Biol. Chem.* 278, 2750-2757 10.1074/jbc.M209256200 12429738

[DMM017046C21] Jensen S. A., Reinhardt D. P., Gibson M. A. and Weiss A. S. (2001). Protein interaction studies of MAGP-1 with tropoelastin and fibrillin-1. *J. Biol. Chem.* 276, 39661-39666 10.1074/jbc.M104533200 11481325

[DMM017046C22] Keene D. R., Maddox B. K., Kuo H. J., Sakai L. Y. and Glanville R. W. (1991). Extraction of extendable beaded structures and their identification as fibrillin-containing extracellular matrix microfibrils. *J. Histochem. Cytochem.* 39, 441-449 10.1177/39.4.2005373 2005373

[DMM017046C23] Kinsey R., Williamson M. R., Chaudhry S., Mellody K. T., McGovern A., Takahashi S., Shuttleworth C. A. and Kielty C. M. (2008). Fibrillin-1 microfibril deposition is dependent on fibronectin assembly. *J. Cell Sci.* 121, 2696-2704 10.1242/jcs.029819 18653538

[DMM017046C24] Koo B.-H., Le Goff C., Jungers K. A., Vasanji A., O'Flaherty J., Weyman C. M. and Apte S. S. (2007). ADAMTS-like 2 (ADAMTSL2) is a secreted glycoprotein that is widely expressed during mouse embryogenesis and is regulated during skeletal myogenesis. *Matrix Biol.* 26, 431-441 10.1016/j.matbio.2007.03.003 17509843

[DMM017046C25] Kutz W. E., Wang L. W., Bader H. L., Majors A. K., Iwata K., Traboulsi E. I., Sakai L. Y., Keene D. R. and Apte S. S. (2011). ADAMTS10 protein interacts with Fibrillin-1 and promotes its deposition in extracellular matrix of cultured fibroblasts. *J. Biol. Chem.* 286, 17156-17167 10.1074/jbc.M111.231571 21402694 PMC3089559

[DMM017046C26] Le Goff C. and Cormier-Daire V. (2009). Genetic and molecular aspects of acromelic dysplasia. *Pediatr. Endocrinol. Rev.* 6, 418-423. 19396027

[DMM017046C27] Le Goff C., Morice-Picard F., Dagoneau N., Wang L. W., Perrot C., Crow Y. J., Bauer F., Flori E., Prost-Squarcioni C., Krakow D. et al. (2008). ADAMTSL2 mutations in geleophysic dysplasia demonstrate a role for ADAMTS-like proteins in TGF-beta bioavailability regulation. *Nat. Genet.* 40, 1119-1123 10.1038/ng.199 18677313 PMC2675613

[DMM017046C28] Le Goff C., Mahaut C., Wang L. W., Allali S., Abhyankar A., Jensen S., Zylberberg L., Collod-Beroud G., Bonnet D., Alanay Y. et al. (2011). Mutations in the TGFbeta binding-protein-like domain 5 of FBN1 are responsible for acromicric and geleophysic dysplasias. *Am. J. Hum. Genet.* 89, 7-14 10.1016/j.ajhg.2011.05.012 21683322 PMC3135800

[DMM017046C29] Lemaire R., Farina G., Kissin E., Shipley J. M., Bona C., Korn J. H. and Lafyatis R. (2004). Mutant fibrillin 1 from tight skin mice increases extracellular matrix incorporation of microfibril-associated glycoprotein 2 and type I collagen. *Arthritis Rheum.* 50, 915-926 10.1002/art.20053 15022335

[DMM017046C30] Lin G., Tiedemann K., Vollbrandt T., Peters H., Bätge B., Brinckmann J. and Reinhardt D. P. (2002). Homo- and heterotypic fibrillin-1 and -2 interactions constitute the basis for the assembly of microfibrils. *J. Biol. Chem.* 277, 50795-50804 10.1074/jbc.M210611200 12399449

[DMM017046C31] Mariencheck M. C., Davis E. C., Zhang H., Ramirez F., Rosenbloom J., Gibson M. A., Parks W. C. and Mecham R. P. (1995). Fibrillin-1 and fibrillin-2 show temporal and tissue-specific regulation of expression in developing elastic tissues. *Connect. Tissue Res.* 31, 87-97 10.3109/03008209509028396 15612324

[DMM017046C32] Massam-Wu T., Chiu M., Choudhury R., Chaudhry S. S., Baldwin A. K., McGovern A., Baldock C., Shuttleworth C. A. and Kielty C. M. (2010). Assembly of fibrillin microfibrils governs extracellular deposition of latent TGF beta. *J. Cell Sci.* 123, 3006-3018 10.1242/jcs.073437 20699357 PMC2923573

[DMM017046C33] McCulloch D. R., Goff C. L., Bhatt S., Dixon L. J., Sandy J. D. and Apte S. S. (2009). Adamts5, the gene encoding a proteoglycan-degrading metalloprotease, is expressed by specific cell lineages during mouse embryonic development and in adult tissues. *Gene Expr. Patterns* 9, 314-323 10.1016/j.gep.2009.02.006 19250981 PMC2725439

[DMM017046C34] McGowan S. E., Holmes A. J., Mecham R. P. and Ritty T. M. (2008). Arg-Gly-Asp-containing domains of fibrillins-1 and -2 distinctly regulate lung fibroblast migration. *Am. J. Respir. Cell Mol. Biol.* 38, 435-445 10.1165/rcmb.2007-0281OC 18006876

[DMM017046C35] Mellersh C. (2012). DNA testing and domestic dogs. *Mamm. Genome* 23, 109-123 10.1007/s00335-011-9365-z 22071879 PMC3275738

[DMM017046C36] Nakanishi H., Sugiura T., Streisand J. B., Lonning S. M. and Roberts J. D. Jr (2007). TGF-beta-neutralizing antibodies improve pulmonary alveologenesis and vasculogenesis in the injured newborn lung. *Am. J. Physiol. Lung Cell. Mol. Physiol.* 293, L151-L161 10.1152/ajplung.00389.2006 17400601

[DMM017046C37] Neptune E. R., Frischmeyer P. A., Arking D. E., Myers L., Bunton T. E., Gayraud B., Ramirez F., Sakai L. Y. and Dietz H. C. (2003). Dysregulation of TGF-beta activation contributes to pathogenesis in Marfan syndrome. *Nat. Genet.* 33, 407-411 10.1038/ng1116 12598898

[DMM017046C38] Nistala H., Lee-Arteaga S., Smaldone S., Siciliano G. and Ramirez F. (2010a). Extracellular microfibrils control osteoblast-supported osteoclastogenesis by restricting TGF{beta} stimulation of RANKL production. *J. Biol. Chem.* 285, 34126-34133 10.1074/jbc.M110.125328 20729550 PMC2962511

[DMM017046C39] Nistala H., Lee-Arteaga S., Smaldone S., Siciliano G., Carta L., Ono R. N., Sengle G., Arteaga-Solis E., Levasseur R., Ducy P. et al. (2010b). Fibrillin-1 and -2 differentially modulate endogenous TGF-beta and BMP bioavailability during bone formation. *J. Cell Biol.* 190, 1107-1121 10.1083/jcb.201003089 20855508 PMC3101602

[DMM017046C40] Panagopoulos P., Fryssira H., Koutras I., Daskalakis G., Economou A., Benetou V. and Antsaklis A. (2005). Geleophysic dysplasia: a patient with a severe form of the disorder. *J. Obstet. Gynaecol.* 25, 818-820 10.1080/01443610500336058 16368598

[DMM017046C41] Paoletti L. C., Pinel J., Kennedy R. C. and Kasper D. L. (2000). Maternal antibody transfer in baboons and mice vaccinated with a group B streptococcal polysaccharide conjugate. *J. Infect. Dis.* 181, 653-658 10.1086/315285 10669351

[DMM017046C42] Penner A. S., Rock M. J., Kielty C. M. and Shipley J. M. (2002). Microfibril-associated glycoprotein-2 interacts with fibrillin-1 and fibrillin-2 suggesting a role for MAGP-2 in elastic fiber assembly. *J. Biol. Chem.* 277, 35044-35049 10.1074/jbc.M206363200 12122015

[DMM017046C43] Pentsuk N. and van der Laan J. W. (2009). An interspecies comparison of placental antibody transfer: new insights into developmental toxicity testing of monoclonal antibodies. *Birth Defects Res. B Dev. Reprod. Toxicol.* 86, 328-344 10.1002/bdrb.20201 19626656

[DMM017046C44] Pessier A. P. and Potter K. A. (1996). Ocular pathology in bovine Marfan's syndrome with demonstration of altered fibrillin immunoreactivity in explanted ciliary body cells. *Lab. Invest.* 75, 87-95. 8683943

[DMM017046C45] Piha-Gossack A., Sossin W. and Reinhardt D. P. (2012). The evolution of extracellular fibrillins and their functional domains. *PLoS ONE* 7, e33560 10.1371/journal.pone.0033560 22438950 PMC3306419

[DMM017046C46] Pontz B. F., Stoss H., Henschke F., Freisinger P., Karbowski A. and Spranger J. (1996). Clinical and ultrastructural findings in three patients with geleophysic dysplasia. *Am. J. Med. Genet.* 63, 50-54 10.1002/(SICI)1096-8628(19960503)63:1<50::AID-AJMG11>3.0.CO;2-T 8723086

[DMM017046C47] Qi Z., Zhao H., Zhang Q., Bi Y., Ren L., Zhang X., Yang H., Yang X., Wang Q., Li C. et al. (2012). Acquisition of maternal antibodies both from the placenta and by lactation protects mouse offspring from Yersinia pestis challenge. *Clin. Vaccine Immunol.* 19, 1746-1750 10.1128/CVI.00455-12 22933398 PMC3491559

[DMM017046C48] Robinson P. N., Arteaga-Solis E., Baldock C., Collod-Beroud G., Booms P., De Paepe A., Dietz H. C., Guo G., Handford P. A., Judge D. P. et al. (2006). The molecular genetics of Marfan syndrome and related disorders. *J. Med. Genet.* 43, 769-787 10.1136/jmg.2005.039669 16571647 PMC2563177

[DMM017046C49] Sabatier L., Chen D., Fagotto-Kaufmann C., Hubmacher D., McKee M. D., Annis D. S., Mosher D. F. and Reinhardt D. P. (2009). Fibrillin assembly requires fibronectin. *Mol. Biol. Cell* 20, 846-858 10.1091/mbc.E08-08-0830 19037100 PMC2633374

[DMM017046C50] Saito M., Kurokawa M., Oda M., Oshima M., Tsutsui K., Kosaka K., Nakao K., Ogawa M., Manabe R.-i., Suda N. et al. (2011). ADAMTSL6beta protein rescues fibrillin-1 microfibril disorder in a Marfan syndrome mouse model through the promotion of fibrillin-1 assembly. *J. Biol. Chem.* 286, 38602-38613 10.1074/jbc.M111.243451 21880733 PMC3207443

[DMM017046C51] Sakamoto H., Broekelmann T., Cheresh D. A., Ramirez F., Rosenbloom J. and Mecham R. P. (1996). Cell-type specific recognition of RGD- and non-RGD-containing cell binding domains in fibrillin-1. *J. Biol. Chem.* 271, 4916-4922 10.1074/jbc.271.9.4916 8617764

[DMM017046C52] Santolaya J. M., Groninga L. C., Delgado A., Monasterio J. L., Camarero C. and Bilbao F. J. (1997). Patients with geleophysic dysplasia are not always geleophysic. *Am. J. Med. Genet.* 72, 85-90 10.1002/(SICI)1096-8628(19971003)72:1<85::AID-AJMG18>3.0.CO;2-Q 9295082

[DMM017046C53] Segade F., Broekelmann T. J., Pierce R. A. and Mecham R. P. (2000). Revised genomic structure of the human MAGP1 gene and identification of alternate transcripts in human and mouse tissues. *Matrix Biol.* 19, 671-682 10.1016/S0945-053X(00)00115-3 11102756

[DMM017046C54] Segade F., Trask B. C., Broekelmann T. J., Pierce R. A. and Mecham R. P. (2002). Identification of a matrix-binding domain in MAGP1 and MAGP2 and intracellular localization of alternative splice forms. *J. Biol. Chem.* 277, 11050-11057 10.1074/jbc.M110347200 11796718

[DMM017046C55] Sengle G., Charbonneau N. L., Ono R. N., Sasaki T., Alvarez J., Keene D. R., Bachinger H. P. and Sakai L. Y. (2008). Targeting of bone morphogenetic protein growth factor complexes to fibrillin. *J. Biol. Chem.* 283, 13874-13888 10.1074/jbc.M707820200 18339631 PMC2376219

[DMM017046C56] Shi Y., Tu Y., De Maria A., Mecham R. P. and Bassnett S. (2013). Development, composition, and structural arrangements of the ciliary zonule of the mouse. *Invest. Ophthalmol. Vis. Sci.* 54, 2504-2515 10.1167/iovs.13-11619 23493297 PMC3621578

[DMM017046C57] Shohat M., Gruber H. E., Pagon R. A., Witcoff L. J., Lachman R., Ferry D., Flaum E. and Rimoin D. L. (1990). Geleophysic dysplasia: a storage disorder affecting the skin, bone, liver, heart, and trachea. *J. Pediatr.* 117, 227-232 10.1016/S0022-3476(05)80534-7 2380821

[DMM017046C58] Spranger J., Gilbert E. F., Arya S., Hoganson G. M. I. and Opitz J. M. (1984). Geleophysic dysplasia. *Am. J. Med. Genet.* 19, 487-499 10.1002/ajmg.1320190310 6507495

[DMM017046C59] Taipale J., Saharinen J., Hedman K. and Keski-Oja J. (1996). Latent transforming growth factor-beta 1 and its binding protein are components of extracellular matrix microfibrils. *J. Histochem. Cytochem.* 44, 875-889 10.1177/44.8.8756760 8756760

[DMM017046C60] Tiedemann K., Batge B., Muller P. K. and Reinhardt D. P. (2001). Interactions of fibrillin-1 with heparin/heparan sulfate, implications for microfibrillar assembly. *J. Biol. Chem.* 276, 36035-36042 10.1074/jbc.M104985200 11461921

[DMM017046C61] Traboulsi E. I., Whittum-Hudson J. A., Mir S. and Maumenee I. H. (2000). Microfibril abnormalities of the lens capsule in patients with Marfan syndrome and ectopia lentis. *Ophthalmic Genet.* 21, 9-15 10.1076/1381-6810(200003)2111-IFT009 10779844

[DMM017046C62] Trask B. C., Broekelmann T., Ritty T. M., Trask T. M., Tisdale C. and Mecham R. P. (2001). Posttranslational modifications of microfibril associated glycoprotein-1 (MAGP-1). *Biochemistry* 40, 4372-4380 10.1021/bi002738z 11284693

[DMM017046C63] Weinbaum J. S., Broekelmann T. J., Pierce R. A., Werneck C. C., Segade F., Craft C. S., Knutsen R. H. and Mecham R. P. (2008). Deficiency in microfibril-associated glycoprotein-1 leads to complex phenotypes in multiple organ systems. *J. Biol. Chem.* 283, 25533-25543 10.1074/jbc.M709962200 18625713 PMC2533084

[DMM017046C64] Werneck C. C., Trask B. C., Broekelmann T. J., Trask T. M., Ritty T. M., Segade F. and Mecham R. P. (2004). Identification of a major microfibril-associated glycoprotein-1-binding domain in fibrillin-2. *J. Biol. Chem.* 279, 23045-23051 10.1074/jbc.M402656200 15044481

[DMM017046C65] Wheatley H. M., Traboulsi E. I., Flowers B. E., Maumenee I. H., Azar D., Pyeritz R. E. and Whittum-Hudson J. A. (1995). Immunohistochemical localization of fibrillin in human ocular tissues. Relevance to the Marfan syndrome. *Arch. Ophthalmol.* 113, 103-109 10.1001/archopht.1995.01100010105028 7826283

[DMM017046C66] Zhang H., Apfelroth S. D., Hu W., Davis E. C., Sanguineti C., Bonadio J., Mecham R. P. and Ramirez F. (1994). Structure and expression of fibrillin-2, a novel microfibrillar component preferentially located in elastic matrices. *J. Cell Biol.* 124, 855-863 10.1083/jcb.124.5.855 8120105 PMC2119952

[DMM017046C67] Zhang H., Hu W. and Ramirez F. (1995). Developmental expression of fibrillin genes suggests heterogeneity of extracellular microfibrils. *J. Cell Biol.* 129, 1165-1176 10.1083/jcb.129.4.1165 7744963 PMC2120487

[DMM017046C68] Zilberberg L., Todorovic V., Dabovic B., Horiguchi M., Couroussé T., Sakai L. Y. and Rifkin D. B. (2012). Specificity of latent TGF-beta binding protein (LTBP) incorporation into matrix: role of fibrillins and fibronectin. *J. Cell. Physiol.* 227, 3828-3836 10.1002/jcp.24094 22495824 PMC3404192

