# Supplementary material for: Adamtsl2 deletion results in bronchial fibrillin microfibril accumulation and bronchial epithelial dysplasia – a novel mouse model providing insights into geleophysic dysplasia
Source: Dis Model Mech. 2015 May 1;8(5):487–99. doi: 10.1242/dmm.017046 (PMC4415891; doi:10.1242/dmm.017046)
Supplement: Supplementary Material [file supp_8_5_487__index.html]

Adamtsl2 deletion results in bronchial fibrillin microfibril accumulation and bronchial epithelial dysplasia – a novel mouse model providing insights into geleophysic dysplasia — Supplementary Material 

# *Adamtsl2* deletion results in bronchial fibrillin microfibril accumulation and bronchial epithelial dysplasia – a novel mouse model providing insights into geleophysic dysplasia

## DMM017046 Supplementary Material

**Files in this Data Supplement:**

- **Supplementary Material**
