# Supplementary material for: Adamtsl2 deletion results in bronchial fibrillin microfibril accumulation and bronchial epithelial dysplasia – a novel mouse model providing insights into geleophysic dysplasia
Source: Dis Model Mech. 2015 May 1;8(5):487–99. doi: 10.1242/dmm.017046 (PMC4415891; doi:10.1242/dmm.017046)
Supplement: Supplementary Material [file supp_8.5.487_DMM017046.pdf]

Supplemental Figure S1: Bronchial epithelial cell polarity and integrity is maintained in *Adamtsl2*-/- bronchi. (A-E) Staining for selected markers of bronchial cell differentiation and intracellular compartments is shown in *Adamtsl2*-/- (-/-) and WT mice. Comparable smooth muscle actin (SMA, from n = 5 per genotype) (A) and pan-cytokeratin (pCK, from n = 2 per genotype) (B) are indicative of an intact smooth muscle cell layer and epithelial cell identity, respectively, in the bronchi of *Adamtsl2*-/- lungs. The occluding vesicles are not positive for lysosomes (Lamp2, from n = 2 per genotype) (C), or the Golgi apparatus marker GM130 (E, green, n = 3 per genotype), but stained for the ER marker KDEL (from n = 3 per genotype) (D). Staining for ciliated cells using β-tubulin (red) shows no differences between WT and *Adamtsl2*-/- lungs. **(F-I)** Markers for spatial relationships of bronchial epithelium show that despite profound epithelial dysplasia in *Adamtsl2*-/- bronchi, the overall bronchial epithelial identity and orientation were maintained (from n = 2 per genotype), although *Adamtsl2-/-* bronchial epithelium shows reduced staining in H and I. Collagen IV staining indicates an intact basement membrane (F, arrows point to the epithelial/SMC junction), cortical actin, stained with phalloidin-FITC, is distributed apically and laterally in WT and *Adamtsl2*-/- bronchial epithelium (G), PKCζ mainly stains the lumenal side of the bronchial epithelium (H). The zona occludens marker ZO-2, even though weaker in the *Adamtsl2*-/- lungs, indicates tight junctions between bronchial epithelial cells (I). Bronchi are outlined with dashed line. Scale bars = 25 μm, if not indicated otherwise. WT, wild-type, -/-, *Adamtsl2*-/-; B, bronchi; BV, blood vessel.

Supplemental Figure S2: No alteration in cell proliferation or apoptosis in the bronchial epithelium of *Adamtsl2*-/- lungs. Cell proliferation (top panels) was analyzed by staining with an antibody against phospho-histone H3, which marks proliferating cells (from n = 2 per

genotype). Positive (proliferating) cells are indicated with arrows. Apoptosis (bottom panels) was assessed with TUNEL staining (from n = 2 per genotype). TUNEL positive cells are indicated with arrows. Scale bars = 25 µm. B, bronchi.

Supplemental Figure S3: Immunolocalization of FBN1 in paraffin-embedded lung sections. FBN1 predominantly localizes to the blood vessel walls and only a minor amount is found in the bronchial epithelium at E14.5 (from n = 3 per genotype), E17.5 (from n = 3 per genotype), and P0 (from n > 6 per genotype). Nuclei are counterstained with DAPI (blue). Quantification of mean integrated density of fluorescence signal for FBN1 showed no statistically significant difference between the WT and *Adamtsl2*-/- lungs. Scale bars = 25 µm. B, bronchi; asterisk, blood vessel.

Supplemental Figure S4: Non-canonical TGFβ-signaling pathways are not altered in *Adamtsl2*-/- lungs. (A) Western blots are shown for pErk1/2 or pp38 and total Erk1 or p38, respectively, using total protein extracted from *Adamtsl2*-/- or WT lungs at E17.5 (left, n = 1 for WT, n = 4 for *Adamtsl2*-/-; n = 4 for *Adamtsl2*+/-) and P0 (right, n = 3 for WT and *Adamtsl2*-/-; n = 2 for *Adamtsl2*+/-). Equal amounts of protein were loaded and the mean intensity of the pErk1/2 or pp38 bands was quantified using ImageJ and normalized to the amount of total Erk1or total p38, respectively. **(B)** Absence of nuclear pSmad2 staining in NAB treated lungs compared to IgG treated lungs is indicative of the efficiency of the NAB for blocking TGFβ signaling (from n = 2 per genotype). Scale bars = 25 µm.

Supplemental Table S1. Frequencies of genotypes arising from *Adamtsl2+/-* intercrosses[1]

	Genotype		
**Stage**	*Adamtsl2+/+*	*Adamtsl2+/-*	*Adamtsl2-/-*
E14.5 – E18.5	33 (35.1)	34 (36.2)	27 (28.7)
Postnatal day 0 - 1	30 (33.3)	35 (38.9)	25 (27.8)[2]
Postnatal day 7	60 (37.5)	95 (59.4)[4]	5 (3.1)[3]
Shown are the number of mice (percentage) at the indicated ages			

[1]Numbers are combined from intercrosses arising from *Adamtsl2*+/- mice before and after *Neo* deletion.

[2]8/21 (38.1%) were found gasping and/or cyanotic, others were found dead.

[3]These mice were small, with tight skin and joint contractures, and died before 14 days of age.

[4]These mice were viable, fertile and most were indistinguishable from WT littermates. A few *Adamtsl2+/-* mice were small, with variably tight skin and excess collagen deposition around bronchi.

Supplemental Table S2. Primers used for genotyping and qRT-PCR

Primer	***Sequence (5'-3')***	***Application***
Adamtsl2 WT F	GTACCAGCTCTGCAGAGTGC	Genotyping
Adamtsl2 WT R	AAGCTCCTCCCATCCGGTGG	Genotyping
Adamtsl2 KO F	AGCTGCGTGTTGTCTCCCC	Genotyping
Adamtsl2 KO R	CACTGAGTCTCTGGCATCTC	Genotyping
Adamtsl2 Exon2/3 F	GCTGTAGCAGTTGTGGCT	qRT-PCR
Adamtsl2 Exon2/3 R	CCTCTAGGCTGTTGGATGTG	qRT-PCR
Adamtsl2 Exon 8/9 F	CCAGATTGTGGAGAGGAAGAAG	qRT-PCR
Adamtsl2 Exon 8/9 R	GTCCACTTTGTAGTTGCCATTG	qRT-PCR
Fbn1 F	GCCAGAAAGGGTACATCGG	qRT-PCR
Fbn1 R	ACACACCTCCCTCCGTT	qRT-PCR
Fbn2 F	GTGAAACCACACAGAAATGTGAA	qRT-PCR
Fbn2 R	GAACAGTCGCCAGTCTCAC	qRT-PCR
Fn F	GTCTAGGCGAAGGCAATGG	qRT-PCR
Fn R	CCTATAGGATGTCCGGGTGT	qRT-PCR
Magp1 F	CATCCACAAGCCTTGCAAAC	qRT-PCR
Magp1 R	CAGACAGTGCGGACACATATT	qRT-PCR
Ltbp1 F	GGTTATTTGCCATCTTCCGTGTA	qRT-PCR
Ltbp1 R	GAAATTTGGAGGGCACTGACA	qRT-PCR
m*Hprt1* F	GATCCATTCCTATGACTGTAGAT	qRT-PCR
m*Hprt1* R	AGATCATCTCCACCAATAACTT	qRT-PCR
m*Gapd* F	GTGCTGAGTATGTCGTGGAG	qRT-PCR
m*Gapd* R	GCGGAGATGATGACCCTTT	qRT-PCR

Supplemental Table S3. Antibodies used for immunostaining and western blotting

Antibody	*Host*	*Supplier*	*Product number*	*Dilution*	*Application (Embedding)*	*Antigen retrieval*
α-smooth muscle actin (SMA)	Ms	Sigma	A2547	1:300	IF (P)	-
pan-cytokeratin	Rab	Dako	Z0622	1:200	IF (P)	H/E
KDEL	Ms	ENZO	ADI-SPA-827	1:200	IF (P)	H/E
Magp1	Rab	-	-	1:200	IF (P)	H/E
fibronectin	Rab	Millipore	AB2033	1:200	IF (P)	H/E
fibrillin-1	Rab	-	-	1:250	IF (P, F)	H/E
fibrillin-2	Rab	-	-	1:300	IF (P, F)	H/E
Lamp2	R	Abcam	ABL-93	1:200	IF (F)	-
Ltbp1-K	Rab	-	-	1:250	IF (F)	
GM130	Rab	Abcam	ab52649	1:250	IF (P)	H/E
β-tubulin-IV	Ms	BioGENEX	MU178-US	1:500	IF (P)	- and H/E
Col IV	Rab	Rockland	600-401-106-.01	1:500	IF (P)	proteinase K
Phalloidin-FITC	-	Life Technologies	F432	1:500	IF (F)	-
PKCζ	Ms	Santa Cruz	sc-17781	1:200	IF (P)	-
ZO-2	Rab	Santa Cruz	sc-11448	1:200	IF (P)	-
pSmad2	Rab	Cell Signaling	3101	1:600	IHC (P)	H/E
phospho-histone H3	Rab	Millipore	06-570	1:250	IF	H/E
pSmad2	Rab	Cell Signaling	3101	1:1000	WB	-
Smad2/3	Rab	Cell Signaling	3102	1:1000	WB	-
pSmad1/5/8	Rab	Millipore	AB3848	1:500	WB	-
Smad1	Ms	Millipore	04-1100	1:1000	WB	-
pErk1/2	Rab	Cell Signaling	4376	1:1000	WB	-
Erk1	Rab	Santa Cruz	sc-94	1:1000	WB	-
pp38	Rab	Cell Signaling	4511	1:500	WB	-
p38	Rab	Cell Signaling	9212	1:1000	WB	-
ADAMTSL2 (C3)	Rab	GeneTex	GTX102069	1:500	WB	-

Ms: mouse; *Rab*: rabbit; *R*: rat; *IF:* immunofluorescence, paraffin (P) or frozen (F) sections; *IHC*, immunohistochemistry; *ICC*: immunocytochemistry; *WB*: western blotting; *H/E:* 4 x 1.5 min at 50% power with 30 sec intermission in 10 mM citric acid, 2 mM EDTA, 0.05% Tween-20, pH 6.2, in a microwave oven; *Proteinase K:* 2 min incubation with 20 mg/ml proteinase K solution at room temperature

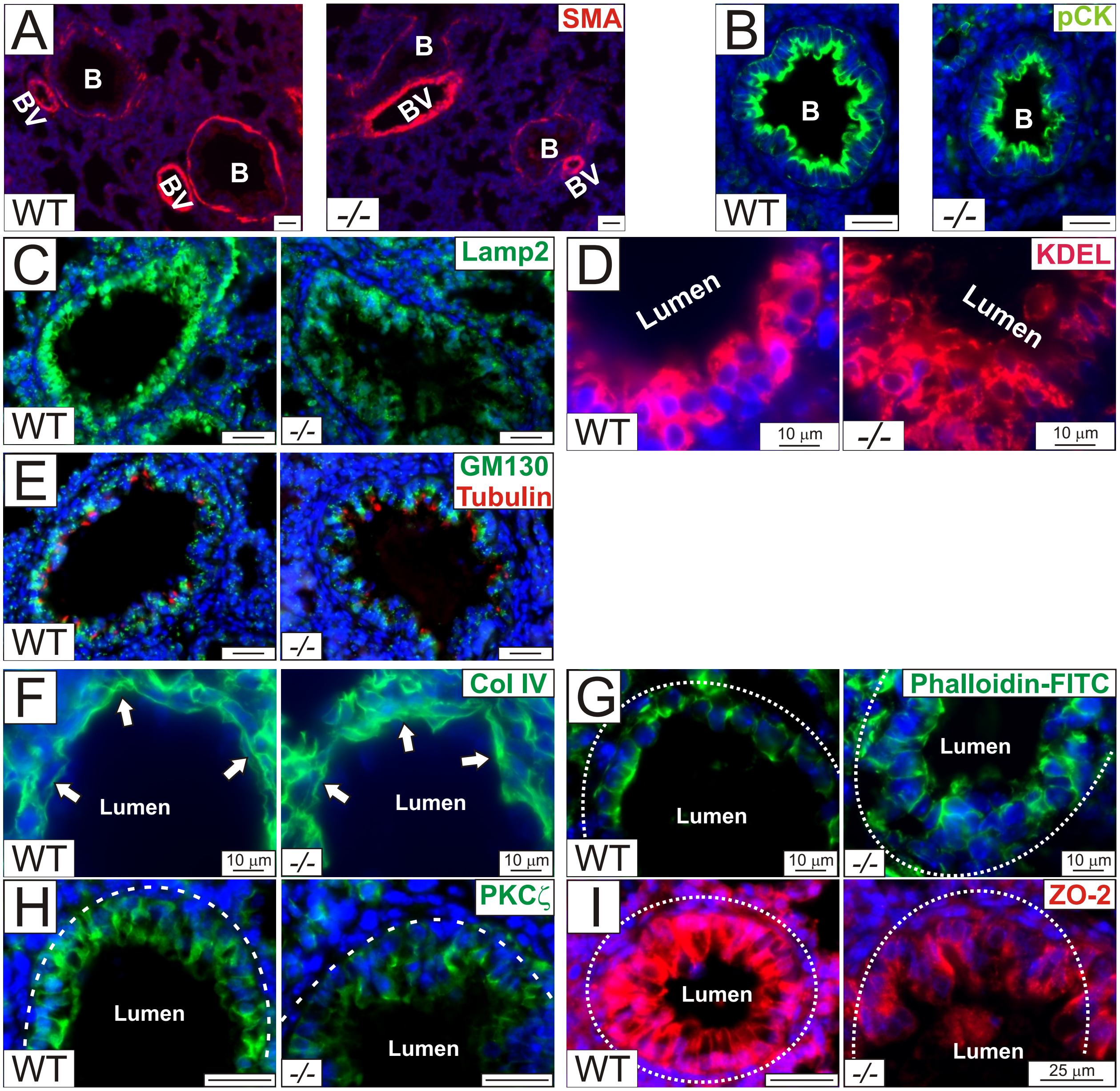

Supplemental Figure S1

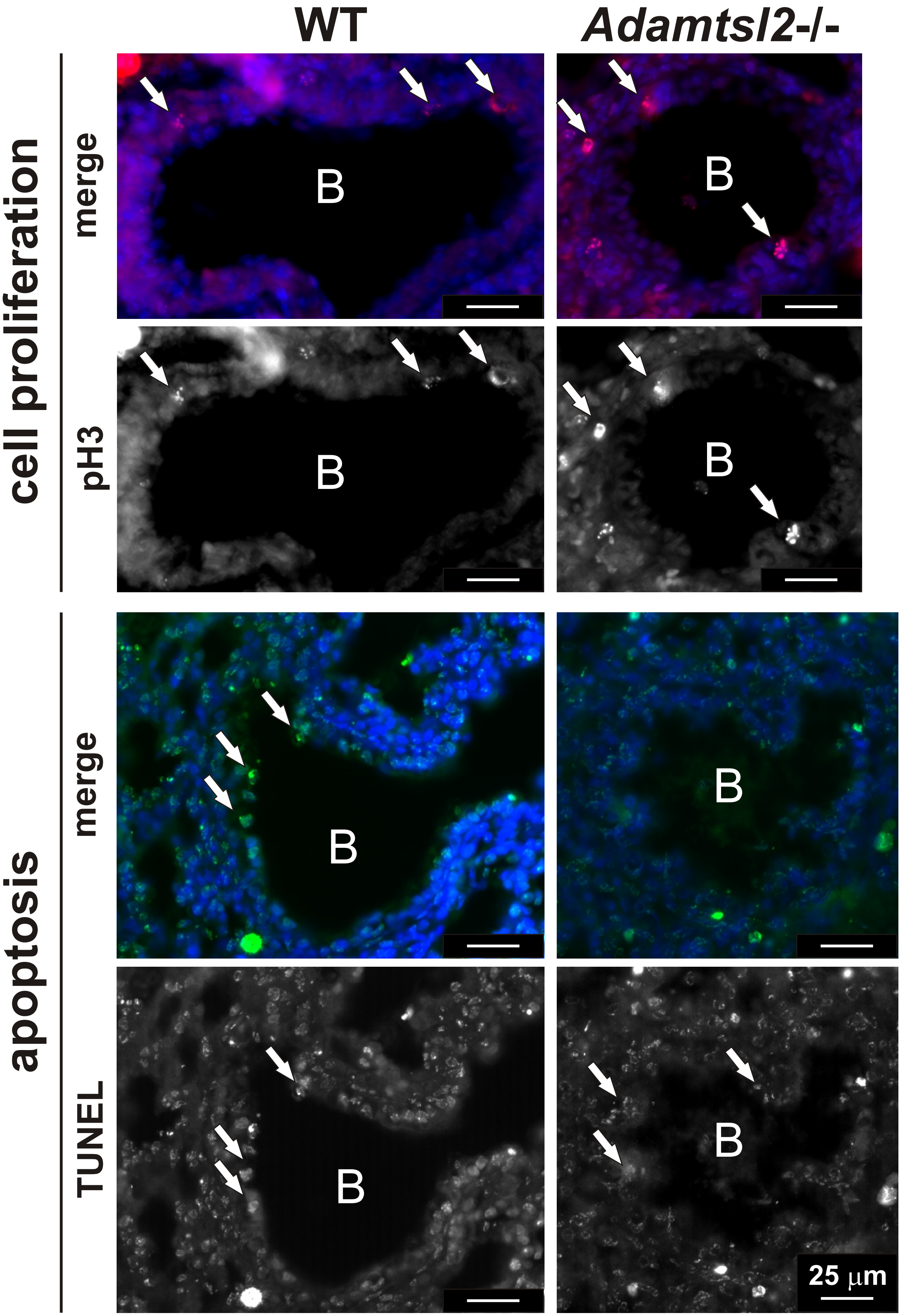

Supplemental Figure S2

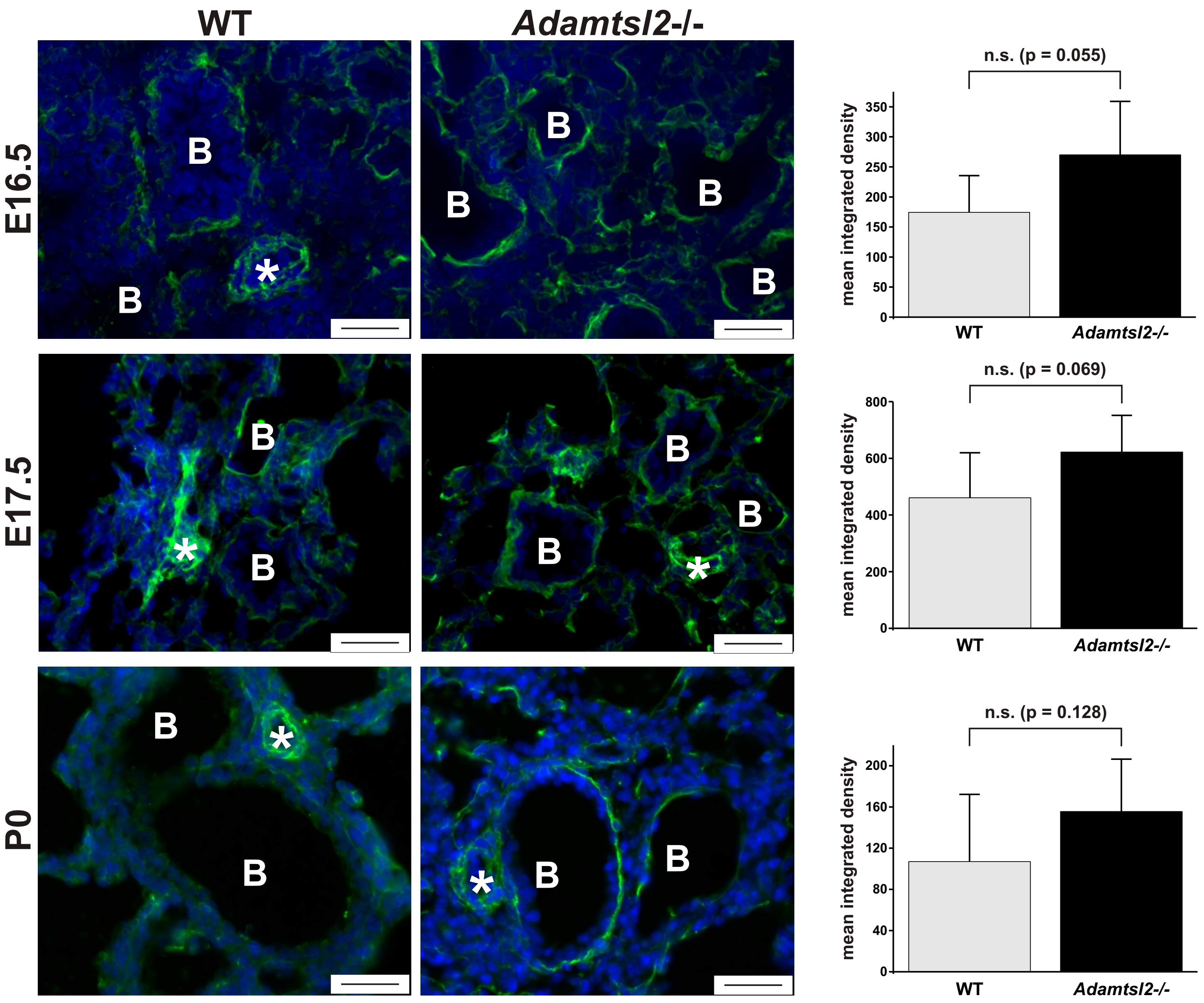

Supplemental Figure S3

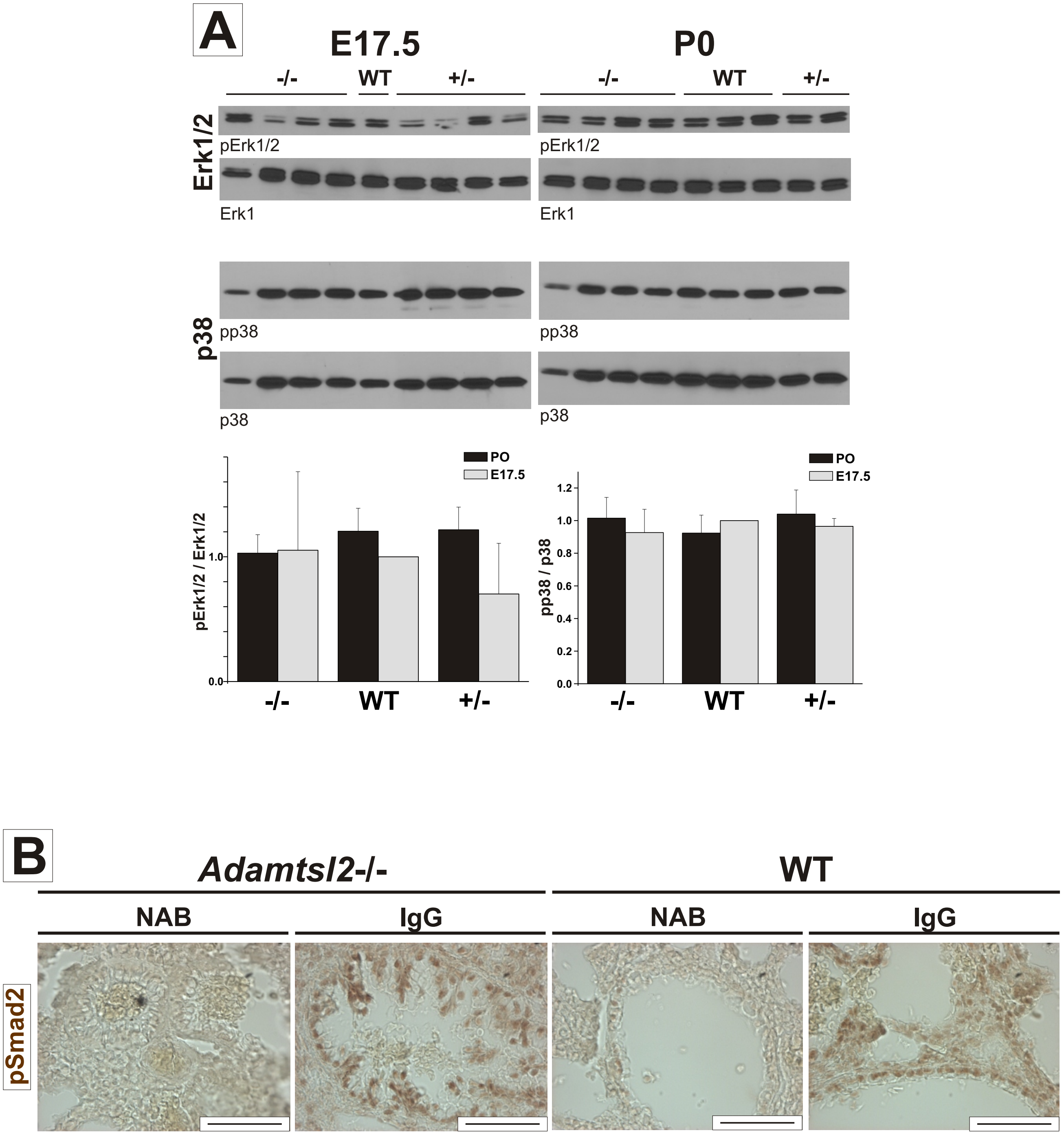

Supplemental Figure S4
